# Thylakoids reorganization enables driving photosynthesis under far‐red light in the microalga *Nannochloropsis gaditana*

**DOI:** 10.1111/nph.70786

**Published:** 2025-12-03

**Authors:** Elisabetta Liistro, Mariano Battistuzzi, Mattia Storti, Beatrice Boccia, Lorenzo Cocola, Giorgio Perin, Tomas Morosinotto, Nicoletta La Rocca

**Affiliations:** ^1^ Department of Biology University of Padova Padova 35131 Italy; ^2^ Institute for Photonics and Nanotechnologies, National Research Council Padova 35131 Italy; ^3^ National Biodiversity Future Center Palermo 90133 Italy

**Keywords:** far‐red photosynthesis, microalgae, *Nannochloropsis gaditana*, photosystems stoichiometry, thylakoids ultrastructure

## Abstract

- Oxygenic photosynthesis is driven by visible light in most photosynthetic organisms, with exceptions in a few cyanobacteria and microalgae species, which can extend the light absorption to far‐red (FR) wavelengths, by synthesizing new pigments or shifting the antennae absorption range beyond 700 nm.
- In this work, we describe a novel mechanism of acclimation in the marine microalga *Nannochloropsis gaditana*, which resulted capable of growth relying solely on FR light. Unexpectedly, the response did not involve the synthesis of red‐shifted absorption forms but a peculiar reorganization of chloroplasts.
- The abundance of photosynthetic complexes changed, with an increased accumulation of all pigment‐binding proteins and photosystem II. Chloroplasts became bigger and thylakoid membranes increased in number, occupying almost all the organelle volume, showing also newly observed structures, composed of a central superstack with perpendicular electron‐dense interconnections, that we propose to name *thylakoidal bodies*.
- To the best of our knowledge, these results describe a novel acclimation strategy to FR light, overall highlighting that the biodiversity of responses to FR light is currently underestimated.

## Introduction

Oxygenic photosynthesis is a fundamental metabolic process that drives primary productivity on Earth, supporting most lifeforms (Raven, 2009). Oxygenic photosynthetic organisms perform photosynthesis through pigment–protein supercomplexes embedded in the thylakoid membrane: photosystems I and II (PSI and PSII). Both photosystems are composed of an antenna system and a reaction center, which respectively harvest and convert light into chemical energy (Bryant & Canniffe, 2018). While reaction centers are highly conserved among photosynthetic organisms and contain Chl*a* as the major pigment together with carotenoids, antenna proteins are more diversified and can host several light‐harvesting pigments depending on the taxon, allowing diverse organisms to tune the absorption of different wavelengths across the visible spectrum (Arshad *et al*., 2022; Lazar *et al*., 2022; La Rocca *et al*., 2024). Most organisms utilize visible light to photosynthesize, while photons of longer wavelengths, such as those in the far‐red (FR) waveband (700–800 nm), are poorly absorbed. However, in the last 30 yr, the long‐wavelength limit of oxygenic photosynthesis has been challenged by the discovery of the ability of different organisms, both prokaryotes and eukaryotes, to use FR light to drive photosynthesis (Elias *et al*., 2024).

*Acaryochloris marina* was the first cyanobacterium discovered to be able to grow using FR light only. This was possible thanks to the constitutive presence of the red‐shifted Chl*d* (λ_max_ = 696 nm in 100% methanol compared to 665 nm of Chl*a*), as the major photosynthetic pigment (Miyashita *et al*., 1996; Loughlin *et al*., 2013; Chen, 2014). Many years later, *Halomicronema hongdechloris* was discovered, a cyanobacterium capable of synthesizing both Chl*d* and the even more red‐shifted Chl*f* (λ_max_ = 707 nm in 100% methanol) (Chen *et al*., 2012; Chen, 2014). Later on, a few different cyanobacteria have been identified to activate a complex acclimation response, termed FR light photoacclimation (FaRLiP), suggesting a peculiar trait spread among cyanobacteria of different genera and ecological niches (Antonaru *et al*., 2025). FaRLiP enables growth under FR light, driving the synthesis of Chl*d*, *f*, FR forms of allophycocyanin, FR paralogs of subunits of PSI, PSII and the phycobilisome and remodeling their structure to absorb FR light (Gan *et al*., 2014, 2015; Gan & Bryant, 2015; Zhao *et al*., 2015). On the contrary, FaRLiP has never been observed in eukaryotic algae nor plants. However, a few eukaryotic phototrophs have been recently found to be able to acclimate to monochromatic red or FR illumination by changing the organization of antenna complexes, resulting in red‐shifted forms of Chl*a* (Wolf & Blankenship, 2019; Elias *et al*., 2024). This relies on the modulation of the electronic environment of Chl*a* bound to antenna complexes, affecting its absorption properties (Morosinotto *et al*., 2003). The specific protein involved in this type of acclimation is variable depending on the species, but the mechanism always requires the synthesis of specific antenna complexes that are able to generate red‐shifted Chl*a* absorption forms which can transfer the excitation energy uphill to a Chl*a*‐containing PSII (Wolf & Blankenship, 2019; Elias *et al*., 2024). Currently, there are only a handful of eukaryotes known to be able to implement this acclimation strategy (Supporting Information Table S1). Among the green eukaryotic algae, the microalgae of the *Ostreobium* genus (Koehne *et al*., 1999; Wilhelm & Jakob, 2006), the recently discovered *Neochloris* sp. Biwa 5‐2 and *Phaeophila dendroides* Sa‐1 (Onami *et al*., 2025; Wang *et al*., 2025) and the macroalga *Prasiola crispa* (Kosugi *et al*., 2020, 2023) are capable of oxygenic photosynthesis in FR light. While in the phylogenetic supergroup that includes Stramenopiles, Alveolates and Rhizarians (SAR supergroup), several microalgae derived from secondary or tertiary endosymbiotic events have been discovered to possess this ability: the diatom *Phaeodactylum tricornutum* (Herbstová *et al*., 2015), the alveolate *Chromera velia* (Bína *et al*., 2014; Kotabová *et al*., 2014) and two eustigmatophytes, FP5 and *Trachydiscus minutus* (Wolf *et al*., 2018; Litvín *et al*., 2019). These microalgae generally inhabit shaded environments enriched in red and FR light, being found as symbionts or free in coastal waters. Still, FR‐utilizing algae from the red lineage (phylum Rhodophyta) have not been discovered yet.

*Nannochloropsis gaditana* (also called *Microchloropsis gaditana*) is an Eustigmatophyte microalga of the SAR supergroup, widely spread in marine waters (Fawley *et al*., 2015). It belongs to the same clade as FP5 and *Trachydiscus minutus*, which can photosynthesize in FR light thanks to red‐shifted antennae (Wolf *et al*., 2018; Litvín *et al*., 2019). As other Eustigmatophytes, *N. gaditana* possesses only Chl*a* and carotenoids, with violaxanthin and vaucheriaxanthin esters being the most abundant carotenoids (Sukenik *et al*., 1992; Lubián *et al*., 2000). *Nannochloropsis gaditana* has also gained ever‐increasing attention as a sustainable cell factory for the production of biofuels, lipids and biomolecules (Ma *et al*., 2016; Hulatt *et al*., 2017; Liu *et al*., 2017).

Interestingly, *N. gaditana* was recently observed to grow under monochromatic FR light (Battistuzzi *et al*., 2023). This was unexpected as a closely related species did not show any apparent red‐shift capability (Litvín *et al*., 2019).

In this work, we investigated the biological basis of FR photon exploitation in *N. gaditana*, using a combination of biochemical, spectroscopic and cell imaging approaches. The results highlight a novel strategy of photo‐acclimation, expanding the current knowledge on the biodiversity of the biological responses to FR light.

## Materials and Methods

### Cultivation conditions

*Nannochloropsis gaditana* (L.M. Lubián), also known as *Microchloropsis gaditana* (L.M. Lubián) M, W. Faawley, I. Jameson & K. P. Fawley CCAP 849/5 (Fawley *et al*., 2015), was obtained from the Culture Collection of Algae and Protozoa (CCAP, United Kingdom). Cultures were grown in sterile F/2 medium (Guillard & Ryther, 1962). Precultures were grown in Erlenmeyer 250 ml flasks under orbital shaking at 100 rpm, in a climatic chamber at 22 ± 1°C under continuous light (TRUE‐LIGHT 18T8, Lightfull) (Zampieri *et al*., 2025) at 25 μmol of photons m^−2^ s^−1^, in the range 380–780 nm. The TRUE‐LIGHT source provides a faithful daylight‐like irradiance; for this reason, from now on, we refer to it as solar‐like (SOL) (Fig. S1). Cultures were kept in the exponential phase by renewing with fresh medium every 3–4 d for at least 2 wk. Cell concentration was measured with a Cellometer Auto X4 cell counter (Nexcelom Bioscience, Lawrence, MA, USA). The starting inoculum was brought to a cell concentration of 10 × 10^6^ cells ml^−1^. In this study, 250 ml flasks containing 50 ml of culture each were prepared with fresh media and transferred under the two light conditions: SOL and FR light. FR light was provided by 730 nm peaked LEDs (SMD OSLON SSL80; Osram Opto Semiconductors, Regensburg, Germany) (Zampieri *et al*., 2025). Each spectrum was supplied as continuous light set at 25 μmol of photons m^−2^ s^−1^, checked through a LI‐COR180 portable spectrometer (LI‐COR180; Li‐Cor, Lincoln, NE, USA). The 730 nm LED emits with a marginal tail at 650–700 nm corresponding to 1.7 μmol of photons m^−2^ s^−1^ of the total 25 μmol of photons m^−2^ s^−1^ provided (Fig. S1). The distribution of the total intensity in the FR, red, green, blue and UV wavebands, in the range 380–780 nm, is reported in Table S2. Temperature and shaking were kept the same as for the precultures. Experiments were conducted on stably acclimated semi‐batch stable cultures, obtained after about a month of periodic refreshing of the flasks to maintain a stable exponential growth phase for both light conditions.

Growth rate was calculated as follows:

(Eqn 1) $$\text{Growth rate}=\frac{\log_{e}\left(n_{f}\right)-\log_{e}\left(n_{i}\right)}{\text{days}}$$

where *n*_*f*_ indicates the cell concentration after the days of growth, and *n*_*i*_ indicates the initial cell concentration.

### *In vivo* absorption, transmittance and reflectance

*In vivo* absorption and transmittance measurements were performed by centrifuging the culture at 1400 ***g*** for 5 min (Sigma Centrifuge 3K15). The supernatant was discarded, and the pellet was homogenized with a pestle. The pellet was then resuspended in 600 μl of fresh F/2 medium and analyzed through a spectrophotometer (Cary 100 UV‐VIS; Agilent, Santa Clara, CA, USA), respectively, in the absorption and transmittance acquisition mode, using optical glass cuvettes, exposing the opaque (opaline) side of the cuvette to the probing ray to correct scattering (Shibata, 1959; Amesz *et al*., 1961; Gan *et al*., 2014).

Reflectance measurements were performed directly on liquid cultures inside a Petri dish. A custom‐made setup was utilized for this measurement (Fig. S2). The device was made by a 3D‐printed scaffold holding an optical fiber, connected to a spectrometer (Flame, OceanOptics, Orlando, FL, USA). The broadband light source for the measurement was provided through a halogen lamp (Halostar Starlite 12V 20WG4; Osram), enabling the calculation of reflectance spectra roughly from 400 to 1000 nm. The light source was put on top of the 3D scaffold, at a distance of *c*. 10 cm and concentrated on the sample by a collecting lens (*f* = 25 mm). Reflectance spectra were recorded via the spectrasuite software (OceanOpticsm). Acquired sample spectra were then normalized against a white reference. This set‐up, implemented from the one published in Battistuzzi *et al*. (2020), is meant to compare the shape of reflectance spectra of different samples. The acquired measurements are not absolute, as the white reference is not calibrated; moreover, the measurements are affected by some stray light coming from the light source and the device structure itself. The comparison of the reflectance spectra, recorded at equal cell concentration, indicates how the reflectance capacity varies at different wavelengths for the two acclimated cultures.

Absorbance, transmittance and reflectance measurements were performed on samples at a final concentration of 50 × 10^6^ cells ml^−1^. Spectra were recorded in the same way for both SOL and FR conditions, allowing for a qualitative comparison to highlight eventual differences in the spectra of acclimated cells (e.g. the appearance of a red shift).

### Pigment extraction and quantification

Chlorophylls (Chls) and Carotenoids (Car) were extracted after centrifuging cells for 10 min at room temperature at 10 000 ***g***. Pellets were resuspended in 100% N,N‐dimethylformamide (DMF) and kept in the dark at 4°C until analysis, for at least 24 h. Extracts were centrifuged at 20 000 ***g*** for 5 min and pigment spectra were recorded using a Cary100 UV‐VIS spectrophotometer (Agilent). Pigments' concentrations were determined using equations for DMF (Wellburn, 1994).

### Spectroscopic analysis

*In vivo* spectroscopic analysis on SOL‐ and FR‐acclimated cells was performed with a Joliot‐type JTS‐10 spectrophotometer (Biologic, Paris, France). The amount of functional photosynthetic complexes was assessed by measuring the electrochromic shift (ECS) spectral change, as the shift in pigment absorption band correlates with changes in the membrane potential (Witt, 1979; Bailleul *et al*., 2010). After 20 min of dark adaptation, intact cells with a final concentration of *c*. 10 μg of Chl ml^−1^ were exposed to a saturating xenon flash. Data were acquired as the difference between signals at 520 and 498 nm (respectively, the positive and negative peaks of the ECS signal in *Nannochloropsis*) (Simionato *et al*., 2013). Kinetic analysis of the ECS signal evidences different phases, the first of which is the ‘fast phase’, which correlates with the charge separation occurring in PSs. Even though we cannot exclude some influence on the ECS signal due to ion concentration across the thylakoid membrane (Cruz *et al*., 2001; Lyu & Lazár, 2022), this method is currently used to highlight variations in the PSs ratio or functionality. PSII contribution was evaluated as the portion of the phase inhibited when cells were poisoned with 3‐(3,4‐dichlorophenyl)‐1,1‐dimethylurea (DCMU; 80 μM) and hydroxylamine (HA; 4 mM), as they block PSII charge separation. PSI contribution was instead evaluated as the portion of the fast phase that was not sensitive to the inhibitors (Bailleul *et al*., 2010; Simionato *et al*., 2011; Perin *et al*., 2017).

Photosynthetic electron flow was evaluated by measuring P_700_ at 705 in intact cells in samples with a final concentration of *c*. 20 μg of Chl ml^−1^. Samples were exposed to a saturating actinic light (630 nm) of 2050 μmol of photons m^−2^ s^−1^ for 15 s in order to follow P_700_ oxidation; light was then switched off to measure rereduction rates of P_700_^+^. Total electron flow (TEF) was derived from the rereduction rates of P_700_^+^ in untreated cells. The contribution of linear electron flow (LEF) and alternative electron flow (AEF) was calculated from the rereduction rates of cells, respectively, poisoned with 80 μM DCMU (blocking the linear flow) and with 80 μM DCMU combined with 300 μM dibromothymoquinone (DBMIB) to block both linear and cyclic flow (Simionato *et al*., 2013; Perin *et al*., 2017).

### Fluorescence measurements

Low temperature (77 K, −196.15°C) fluorescence emission spectra were recorded with a Cary Eclipse Fluorescence Spectrometer (Agilent). 15 × 10^6^ cells were centrifuged at 1500 ***g*** for 10 min at 4°C, and the pellet resuspended in 1 ml of glycerol 60% w/v and Hepes pH 7.5 10 mM. Samples were frozen in liquid nitrogen and kept at −80°C until analysis. Emission spectra were recorded while maintaining the low temperature with liquid nitrogen. Samples were excited at 440 nm, and emission spectra were recorded from 600 to 800 nm.

Chlorophyll fluorescence and photosynthetic parameters *in vivo* were determined on dark‐adapted cells, kept in the dark for 20 min, with a Dual PAM 100 (Heinz‐Walz, Effeltrich, Germany), using a light curve protocol, starting from 0 μmol of photons m^−2^ s^−1^ and increasing light intensity every 1 min up to 2000 μmol of photons m^−2^ s^−1^ in 17 min, followed by 4 min of darkness. The parameters *F*_v_/*F*_m_, NPQ, ΦPSII and qL were obtained with the following equations (Genty *et al*., 1989; Bilger & Björkman, 1990; Kramer *et al*., 2004):

(Eqn 2) $$\frac{F_{V}}{F_{M}}=\frac{F_{M}-F_{0}}{F_{M}}$$

where *F*_0_ is the minimum fluorescence level of dark‐adapted cells, *F*_M_ is the maximum fluorescence level with a saturating light pulse.

(Eqn 3) $$\mathrm{NPQ}=\frac{F_{M}}{F_{M}^{\prime }}-1$$

where $F_{M}^{\prime }$ is the maximum fluorescence level of illuminated cells.

(Eqn 4) $$\text{ΦPSII}=\frac{F_{M}^{\prime }-F}{F_{M}^{\prime }}$$

(Eqn 5) $$\mathrm{qL}=\frac{F_{M}^{\prime }-F}{F_{M}^{\prime }-F_{0}^{\prime }}\cdot \frac{F_{0}^{\prime }}{F}$$

where $F_{0}^{\prime }$ is the minimum fluorescence level of illuminated cells, which was calculated according to Oxborough & Baker (1997), as

(Eqn 6) $$F_{0}^{\prime }=\frac{F_{0}}{\frac{F_{V}}{F_{M}}+\frac{F_{0}}{F_{M}^{\prime }}}$$

The functional antenna size of PSII was measured with a JTS‐10 spectrophotometer in fluorescence mode on cells after 20 min of dark adaptation and 10 min of incubation with DCMU (80 μM) in order to prevent oxidation of Q_A_. Samples with a final concentration of *c*. 15 μg of Chl ml^−1^ were excited with actinic light at 630 nm with an intensity of 320 μmol of photons m^−2^ s^−1^.

### Protein extraction and western blotting analysis

For protein extraction, 500 × 10^6^ of cells were centrifuged for 10 min at 4°C and washed twice with 1 ml of B1 buffer (NaCl 400 mM, MgCl_2_ × 6H_2_O 2 mM, Tricine/KOH pH 7.8 20 mM). Glass beads (acid washed, 150–212 μm; Sigma) were added to the pellet together with 25 μl of B1 buffer supplemented with benzamidine 3 mM, PMSF 1 mM and ε‐amminocaproic acid 4.5 mM. Three cycles of bead beating were executed with a Bullet Blender Storm Pro Homogenizer (Next Advance) for 30 s at maximum speed alternated with 2 min on ice. Sixty microliters of sample buffer (Tris pH 6.8 45 mM, DTT 30 mM, SDS 3% w/v and glycerol 10% w/v), SB hereafter, were added to the lysate and a last cycle of bead beating was performed. Samples were centrifuged at 15 500 ***g*** for 15 min at room temperature, the supernatant was collected in a new tube, and 100 μl of SB was added to the pellet for new bead beating and centrifuging cycles, which were repeated for complete proteome extraction until the pellet was completely white. Chlorophyll content of the extract was quantified in 80% acetone with a Cary100 UV‐VIS spectrophotometer (Agilent); after quantification, samples were incubated for 5 min at 95°C for further protein denaturation. Proteins were separated with SDS‐PAGE, with a stacking gel (Tris pH 6.8125 mM, 4% acrylamide, 0.1% SDS, 0.6% TEMED and 0.1% APS) and a running gel (Tris pH 7.8 1.24 M, 12% acrylamide, 0.33% SDS, 0.7% TEMED and 0.1% APS). Samples were loaded on gels prior to dilution in SB to reach the same Chl concentration for the two experimental conditions; four different Chl concentrations were tested: 0.1, 0.2, 0.5 and 1 μg ml^−1^. Gels were run for 2.5 h at 50 V in Tris pH 8.3250 mM, glycine 1.92 M and 1% SDS. Proteins were transferred to a nitrocellulose membrane (Amersham, Cytiva, Marlborough, MA, USA) in Tris pH 8.3 20 mM, 20% methanol and glycine 152 mM at 100 V for 1 h at 4°C. Membranes were blocked with 10% milk in TBS (Tris pH 7.4 20 mM and NaCl 150 mM) and hybridized with primary antibodies in TTBS (Tris pH 7.4 20 mM, NaCl 150 mM and Tween®20 1%). Membranes were hybridized with either commercial (α‐PsaA 1 : 2000, AS00 6172; Agrisera, Vännäs, Sweden) or polyclonal homemade antibodies (α‐D2 1 : 500, α‐LHCX1 1 : 20000, α‐VCP 1 : 80 000, α‐RbcL 1 : 20 000) produced in rabbit (Perin *et al*., 2015). Membranes were then washed in TTBS and hybridized with the α‐rabbit alkaline phosphatase (AS10 1017; Agrisera) and then washed again in TTBS. The development of membranes was performed with a solution of Tris–HCl pH 9.5100 mM, NaCl 100 mM, MgCl_2_ 5 mM, BCIP (5‐bromo‐4‐chloro‐3′‐indolylphosphate p‐toluidine) 0.165 mg ml^−1^ and NBT (nitro‐blue tetrazolium) 0.675 mg ml^−1^. Bands were then imaged with a CHEMI premium imager (VWR, Milano, Italy) and quantified in Fiji/ImageJ software (National Institutes of Health, Bethesda, MD, USA) with the gel analysis tool.

### Cell imaging: confocal and electron microscopy

For confocal microscopy, cells were immobilized on microscopy slides coated with poly‐lysine (Epredia, Kalamazoo, MI, USA) and live‐imaged with an LSM900 Airyscan2 (Zeiss) confocal microscope. Chlorophyll autofluorescence was used to image chloroplasts. Chlorophyll was excited at 405 nm and fluorescence emission was recorded with a long pass filter transmitting over 655 nm; laser power and gain were kept constant for all imaged samples. For electron microscopy, cells were centrifuged at 3500 ***g*** for 10 min at room temperature, the pellets were fixed overnight at 4°C in 3% glutaraldehyde in sodium cacodylate 0.1 M and postfixed in 1% osmium tetroxide in the same buffer for 2 h. Samples were dehydrated in graded series of ethyl alcohol and propylene oxide and embedded in Epon resin. Ultrathin sections of 80–100 nm were obtained with an ultramicrotome (Ultracut, Reichert‐Jung), stained with uranyl acetate and lead citrate and then analyzed with a transmission electron microscope, TEM, (Tecnai G2, FEI). Image analysis was run using Fiji‐ImageJ (National Institutes of Health). The area of each chloroplast was measured from confocal micrographs, using Chl autofluorescence as a proxy, through the ImageJ ‘analyzing particles’ tool, by setting equal signal thresholds for SOL‐ and FR‐acclimated samples. All other measured parameters were obtained from TEM micrographs. The measured parameters were presence/absence of the *thylakoidal body* (*TB*), the percentage of chloroplast area occupied by thylakoid membranes, the number of membranes per stack, the lumen thickness, the average local thickness of the stacks and the Stacking Repeat Distance (SRD) (Li *et al*., 2020; Mazur *et al*., 2021). For every TEM micrograph, the detection of the *TB* presence or its ongoing formation was evaluated (Fig. S3). The micrographs were considered for analysis only if the thylakoid membranes and eventual *TBs* were sufficiently resolved. The number of membranes per stack was counted through the ‘multi‐point’ ImageJ tool along three transects traced perpendicularly to the major axis of the chloroplast, to represent the variability among cells. The percentage of chloroplasts' area occupied by thylakoid membranes and the average local thickness of the thylakoid stacks were measured through a semi‐automated image processing (Fig. S4) (Martín‐de León *et al*., 2016; Kourra *et al*., 2018). At first, a threshold value, to obtain a binarized image, was applied. The gray values below the set threshold were converted to 0 (corresponding to black, that is, the stroma of the chloroplast) while those above the threshold were converted to 255 (corresponding to white, that is, the thylakoid membrane). Starting from the binarized image, the percentage of area occupied by thylakoid membranes was calculated through the ImageJ ‘analyze particles’ tool as the percentage of white pixels on the total pixels within the chloroplast perimeter. Starting from the same binarized image, the ‘local thickness’ tool calculated the distance in every direction of each white pixel to the closest boundary (a black one). The output of this analysis consists of a local thickness image of the thylakoid membranes (Fig. S4d), coded with a color gradient indicating the width. A histogram of the local thickness of the chloroplast is also obtained, in which the relative frequencies of each thickness value are plotted, with the automatic definition of the bin number and their width. Other calculated parameters are the mode, the mean, the SD of the thickness and the minimum and maximum thickness values.

Finally, for the SRD parameter, the most representative stacks of thylakoids in the cells were chosen, and through the ‘straight’ ImageJ tool their thickness was evaluated in the original image. The SRD value, representing the average thickness of one layer of the stack, composed of the two‐layer photosynthetic membrane, the lumen between them and the neighboring stromal gap (Mazur *et al*., 2021), was calculated as follows:

(Eqn 7) $$\begin{array}{l} SRD\;=\frac{\text{Thylakoid stack thickness}}{\text{number of membranes composing the stack}}. \end{array}$$

The width of each lumen was quantified in the same transects, using the ‘straight’ ImageJ tool.

### Statistical analysis

Statistical analysis was performed with the software graphpad prism v.10.1.0 (GraphPad). Means and SD were calculated for at least four biological replicates per condition. Comparison between the two light conditions was carried out with unpaired *t*‐test or Mann–Whitney test, depending on the dataset, ANOVA analysis (one‐way or two‐way ANOVA, depending on the dataset) and Tukey's multiple comparison tests where needed.

## Results

### *Nannochloropsis gaditana* in FR light does not show red‐shifted absorption

To evaluate the acclimation capabilities to FR light of *N. gaditana*, cultures were cultivated in artificial seawater (F/2), atmospheric CO_2_, with mechanical agitation and under low‐intensity continuous SOL or FR light (25 μmol of photons m^−2^ s^−1^). Cultures were run in semi‐batch mode for a total amount of 200 d (*c*. 6 months) and maintained in the exponential growth phase. This cultivation mode allowed us to obtain stable cultures, fully acclimated to the growth conditions, for the physiological characterization.

**Fig. 1:**
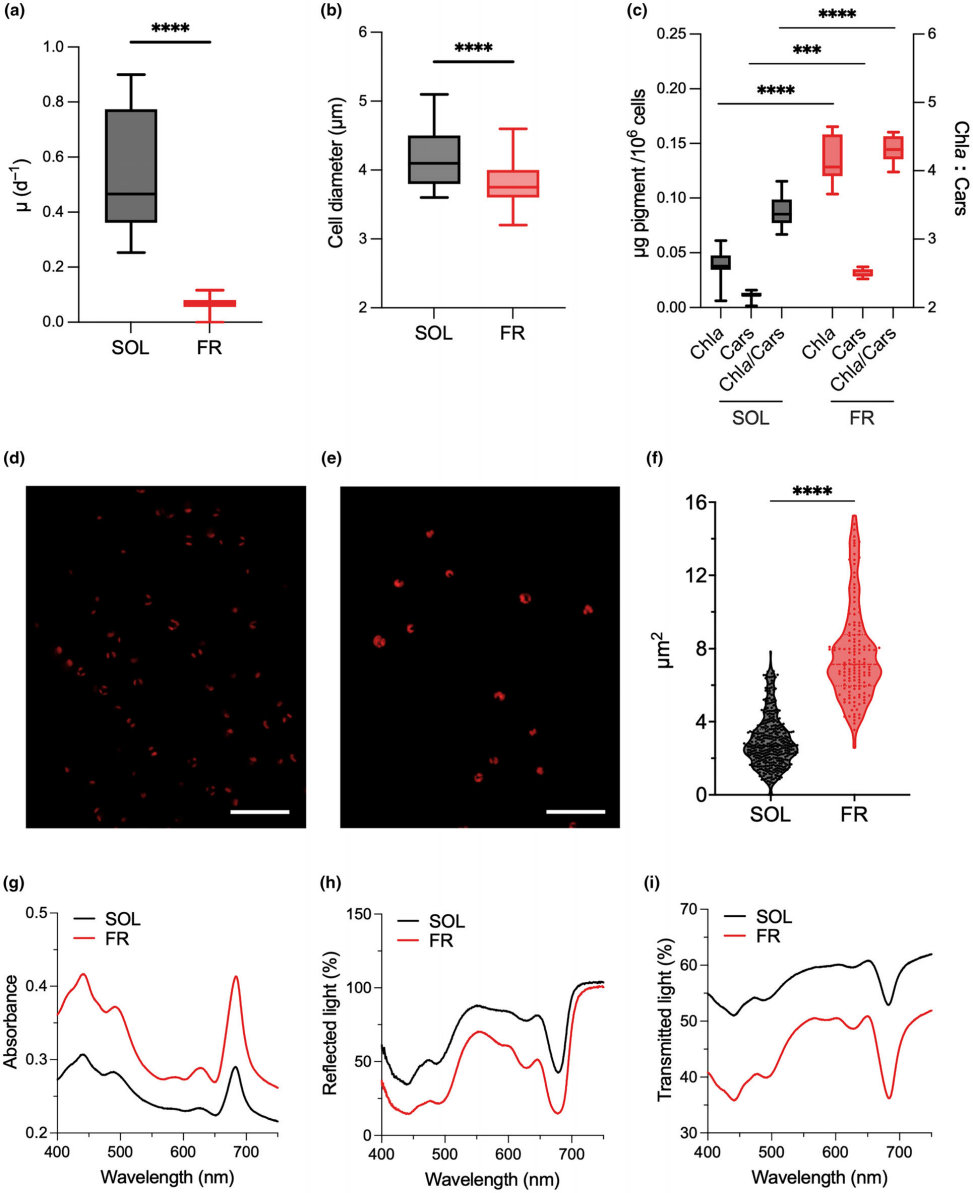
Growth and cell features of *Nannochloropsis gaditana* acclimated to SOL and FR light. (a) Growth rate μ (unpaired *t*‐test, > 100 biological replicates, *P* < 0.0001); (b) cell diameter (μm) (unpaired *t*‐test, > 100 biological replicates, *P* < 0.0001); (c) pigment content of cells in terms of μg of pigments per 10^6^ cells and Chl*a* to carotenoids ratio (two‐way ANOVA, 12 biological replicates, ***, *P* < 0.001; ****, *P* < 0.0001); (d, e) representative confocal images of, respectively, SOL‐acclimated and of FR‐acclimated cells, red color indicates the autofluorescence of Chl*a*; Bars, 20 μm; (f) area (μm^2^) of chlorophyll autofluorescence per cell in SOL‐ and FR‐acclimated cells (unpaired *t*‐test, ≥ 150 cells, *P* < 0.0001). (g) *In vivo* absorption, (h) reflectance and (i) transmission spectra of cultures, measured at equal cell concentrations. Spectra in (g, i) were recorded with a Cary100 spectrophotometer, spectra in (h) were recorded with a custom‐made set‐up. Data in (a, b, c and f) are presented as mean and SD. Reflectance spectra shown in (h) do not represent absolute values. Cars, carotenoids; Chl*a* : Cars, chlorophyll *a* to carotenoids ratio; Chl*a*, chlorophyll *a*; FR, far‐red light; SOL, solar‐like light.

Cells under FR light showed a strongly reduced but significant growth, with a rate of 0.07 ± 0.02 d^−1^ vs the 0.56 ± 0.22 d^−1^ of cells in SOL light (Fig. 1a). FR‐acclimated cells of *N. gaditana* showed a slightly decreased cell diameter (Fig. 1b), and a higher pigment concentration than upon SOL acclimation (Fig. 1c). Chl*a* and total carotenoids in FR were, respectively, 3.4 and 2.7 times higher than in SOL. This was paired with a significant rise in the Chl : car ratio (Fig. 1c), affecting the phenotype of cultures which were darker green in FR light (Fig. S5). Moreover, when observed by confocal imaging, the Chl autofluorescence area, measured as a proxy of the chloroplast area, was significantly larger in FR‐acclimated cells (*P* < 0.0001, Fig. 1d,f) with respect to SOL‐acclimated cells.

To investigate if growth in FR light depended on red‐shifted antennae absorbing beyond 700 nm, the *in vivo* absorption, reflectance and transmittance spectra of the acclimated cells were recorded (Fig. 1g–i). Surprisingly, SOL‐ and FR‐acclimated cells did not show any observable variation in the shape of absorption, reflectance and transmission spectra, and, in particular, they showed unaltered absorption of FR light. Nonetheless, at equal cell concentration, cultures acclimated to FR light showed higher absorption than upon SOL acclimation, due to the higher pigment concentration. Consistent with a higher absorption efficiency, FR‐acclimated cultures showed lower reflectance and transmittance. Also, in those cases, there was no sensible difference in the spectral feature.

### The abundance and functionality of photosynthetic complexes change in FR light

**Fig. 2:**
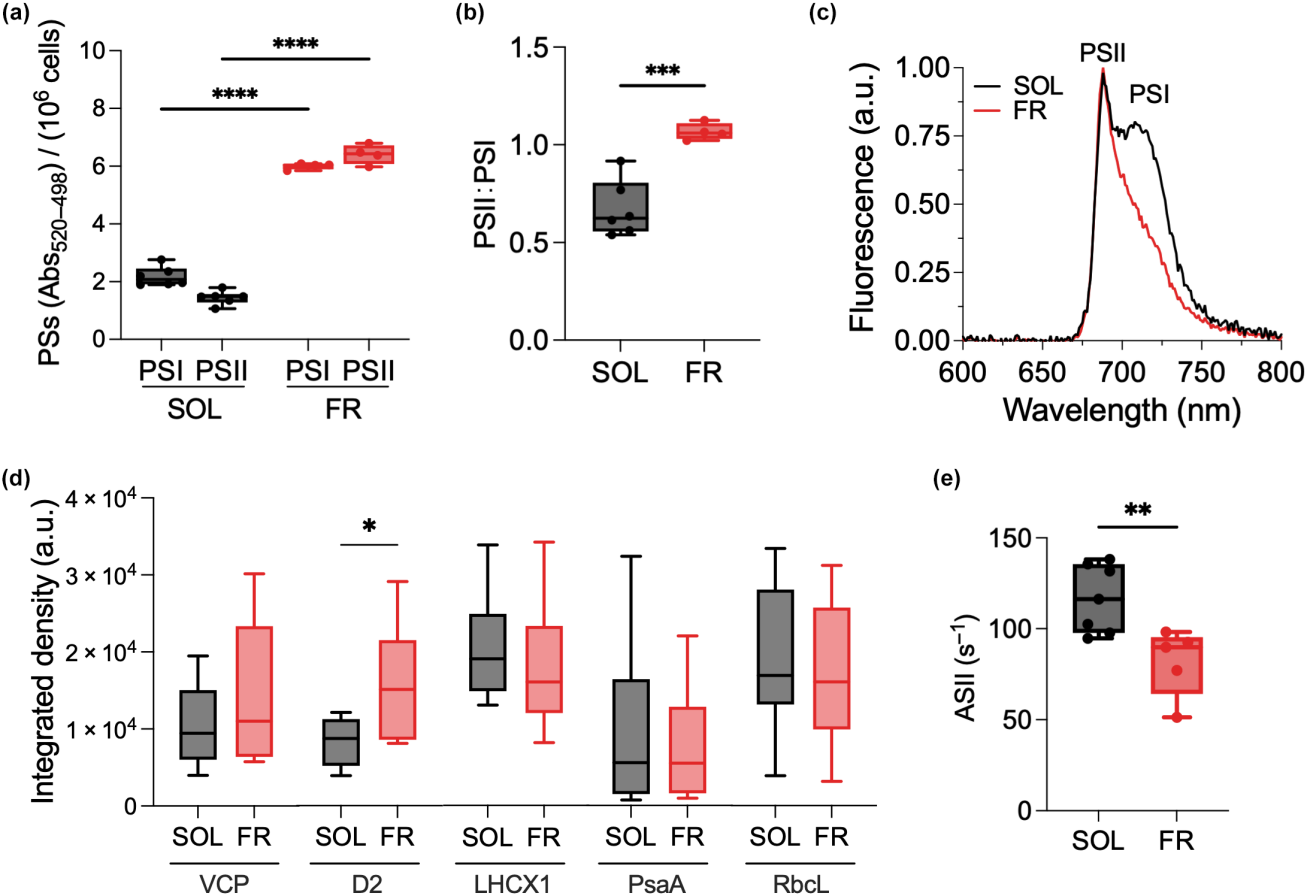
Absorption spectroscopy, fluorescence spectroscopy and biochemical analysis of SOL‐acclimated and FR‐acclimated *Nannochloropsis gaditana* cells. (a) Total photosystem I (PSI) and PSII absorption per 10^6^ cells measured via electrochromic shift (ECS, two‐way ANOVA, ≥ 4 replicates, ****, *P* < 0.0001); (b) PSII : PSI ratio based on ECS signal (unpaired *t*‐test, ≥ 4 replicates, ***, *P* < 0.001); (c) 77 K fluorescence emission spectra, normalized to the PSII maximum chlorophyll fluorescence (688 nm); (d) western blotting analysis using antibodies recognizing VCP, D2, LHCX1, PsaA and RuBisCO large subunit proteins, RbcL, data are presented as the integrated density of bands normalized to the Chl*a* concentration (Mann–Whitney test, eight replicates, *, *P* = 0.0379); (e) functional antenna size of PSII based on room temperature fluorescence kinetics in presence of DCMU (unpaired *t*‐test, ≥ 5 replicates, **, *P* < 0.01). Data in (a, b, d and e) are presented as mean and standard variation. ASII, functional antenna size of photosystem II; D2, photosystem II D2 protein; FR, far‐red light; LHCX1, light‐harvesting complex X1 protein; PsaA, photosystem I P700 Chl*a* apoprotein A1; RbcL, Ribulose‐1,5‐bisphosphate carboxylase/oxygenase large subunit; SOL, solar‐like light.

The impact of acclimation to FR light on the photosynthetic activity of *N. gaditana* was assessed using spectroscopic approaches. Electrochromic shift (ECS) measurements were carried out to evaluate the content of functional photosystems of SOL‐ and FR‐acclimated cells. ECS showed that FR‐acclimated cells had much higher PSI and PSII contents with respect to SOL‐acclimated cells (Fig. 2a), which was consistent with the strong increase in pigment content. Moreover, FR‐acclimated cells showed a significantly higher PSII to PSI ratio (Fig. 2b), suggesting that FR light induced a higher accumulation of the former with respect to PSI in *N. gaditana*. The shift in PSII : PSI ratio was also observed via low‐temperature fluorescence, which allows to distinguish the fluorescence emission of each PS (Lamb *et al*., 2018). In SOL‐acclimated cells, one major peak of emission at 688 nm, attributable to PSII emission and a broad shoulder at 700–740 nm associated with PSI emission were distinguishable (Fig. 2c). In FR‐acclimated cells, instead, only the PSII emission peak was clearly detected, while the PSI emission peak was drastically reduced. This suggests that, consistently with ECS estimation, PSI relative content in FR‐acclimated cells was strongly decreased.

The different content of PSs was confirmed through western blot analysis using antibodies against VCP (the major antenna complex), D2, PsaA, RbcL (RuBisCO Large Subunit) and LHCX1 (Figs 2d, S6). Surprisingly, the analysis did not show major alterations in the relative composition of the photosynthetic apparatus, with the remarkable exception of the D2 protein levels (Fig. 2d), which instead increased in FR‐acclimated cells, confirming the relative increase in PSII that was observed spectroscopically. Antenna proteins VCP and LHCX1 did not match the increase of D2 protein, suggesting fewer antennae per PSII reaction center in FR‐acclimated cells. This was confirmed also by measuring the functional antenna size of PSII via fluorescence kinetics upon illumination in the presence of DCMU (Fig. 2e). Indeed, cells acclimated to FR light had a lower functional PSII antenna size with respect to cells acclimated to SOL light, showing a modulation of the number of antennae in response to FR light.

**Fig. 3:**
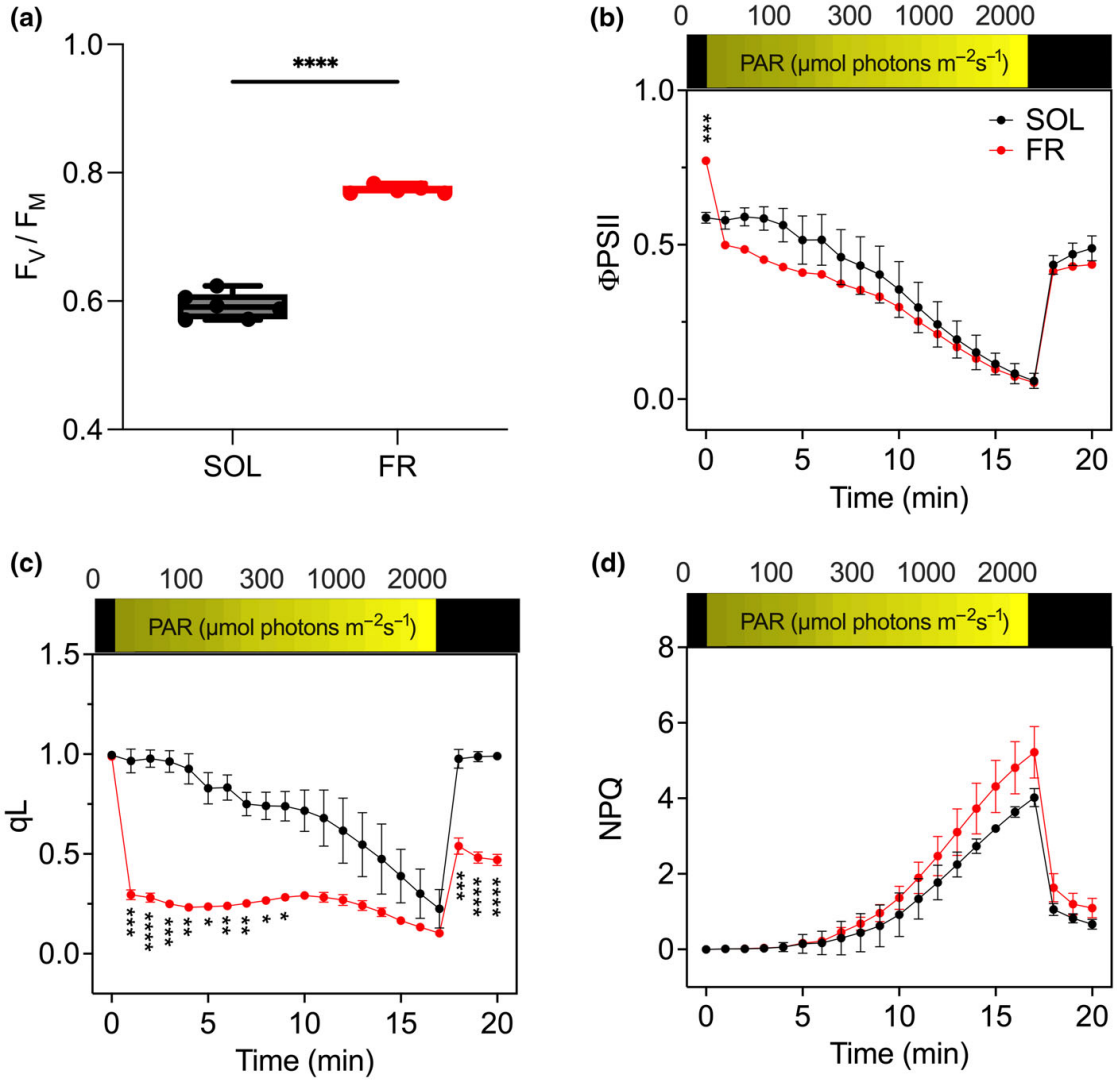
*In vivo* fluorescence measurements of photosystem II (PSII) functionality in SOL‐ and FR‐acclimated *Nannochloropsis gaditana* cells. (a) Maximum quantum yield of PSII (*F*_V_/*F*_M_) upon 20 min of dark acclimation (unpaired *t*‐test, ≤ 5 replicates, *P* < 0.0001), (b) effective photochemical quantum yield of PSII (φPSII), (c) photochemical fluorescence quenching (qL), (d) nonphotochemical quenching (NPQ). Data are presented as mean and SD. In (b–d), two‐way ANOVA, four replicas, *, *P* < 0.05; **, *P* < 0.01; ***, *P* < 0.001; ****, *P* < 0.0001. FR, far‐red light; SOL, solar‐like light; PAR: photosynthetic active radiation.

To assess the implications of this remodeling we evaluated the PSII functionality *in vivo* (Fig. 3). Maximum photochemical quantum yield of PSII (*F*_V_/*F*_M_) was significantly higher in FR‐acclimated cells (Fig. 3a), but, upon illumination, the effective photochemical quantum yield (φPSII) quickly became lower than in SOL‐acclimated cells (Fig. 3b). The saturation of PSII, evaluated with the coefficient of photochemical fluorescence quenching, qL, was in fact reached at much lower light intensities in FR‐acclimated cells than in SOL‐acclimated cells (Fig. 3c). FR‐acclimated cells also showed higher NPQ levels, up to 20% more than upon SOL acclimation (Fig. 3d).

**Fig. 4:**
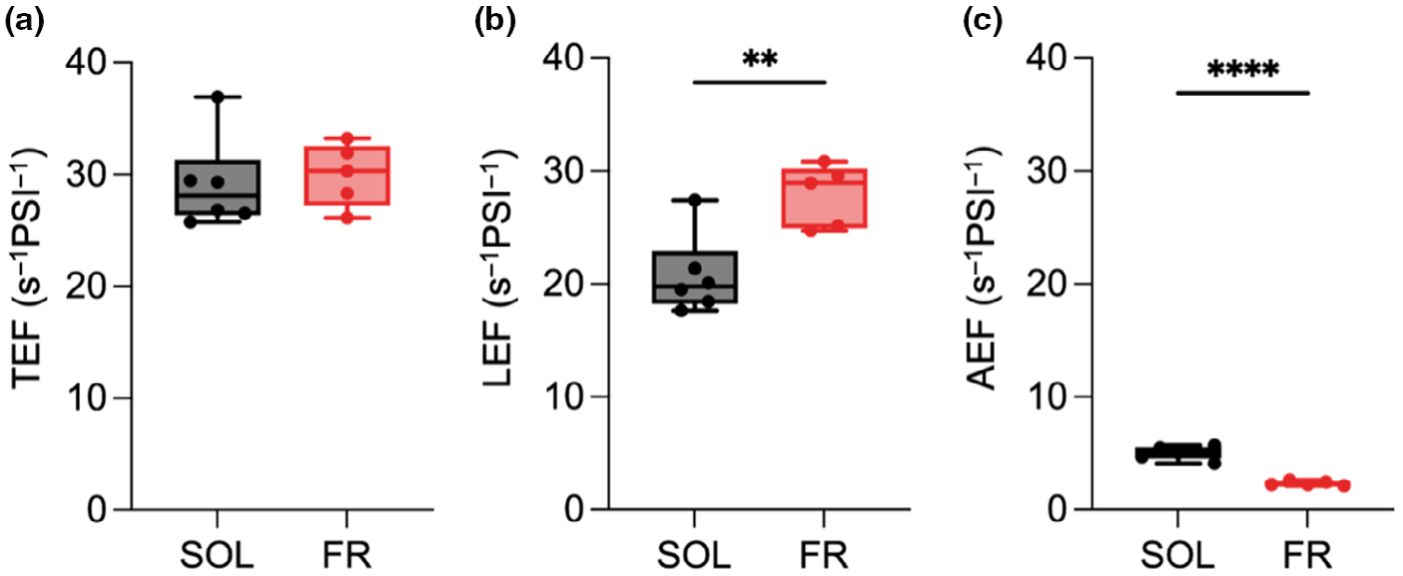
Rates of total (a), linear (b) and alternative (c) electron flow per photosystem I (PSI) in SOL and FR‐acclimated *Nannochloropsis gaditana* cells, measured via rereduction kinetics of PSI in the presence of inhibitors. Data are expressed as mean and SD of at least five biological replicates, unpaired *t*‐test, **, *P* < 0.01; ****, *P* < 0.0001. AEF, alternative electron flow; FR, far‐red light; LEF, linear electron flow; SOL, solar‐like light; TEF, total electron flow.

Given the difference in PSII functionality, time‐resolved absorption spectroscopy was used to assess PSI functionality and evaluate the impact on the total photosynthetic electron flow, as well as the contribution of both linear and alternative electron transports (TEF, LEF and AEF, respectively) in SOL‐ and FR‐acclimated cells (Figs 4, S7). P700 rereduction kinetics after treatment with saturating light were measured in the absence of inhibitors for TEF evaluation, and in the presence of inhibitors (DCMU to inhibit PSII and DBMIB to inhibit cytochrome *b*_*6*_ *f*) for the evaluation of LEF and AEF contribution. SOL‐ and FR‐acclimated cells had comparable TEF rates, but the contribution of LEF and AEF was found to be significantly affected. LEF remained the main component of TEF in both conditions but, in FR‐acclimated cells, LEF was higher than in SOL‐acclimated cells, while AEF contribution decreased.

### FR light triggers an extensive remodeling of chloroplasts ultrastructure, inducing the formation of *thylakoidal bodies*

**Fig. 5:**
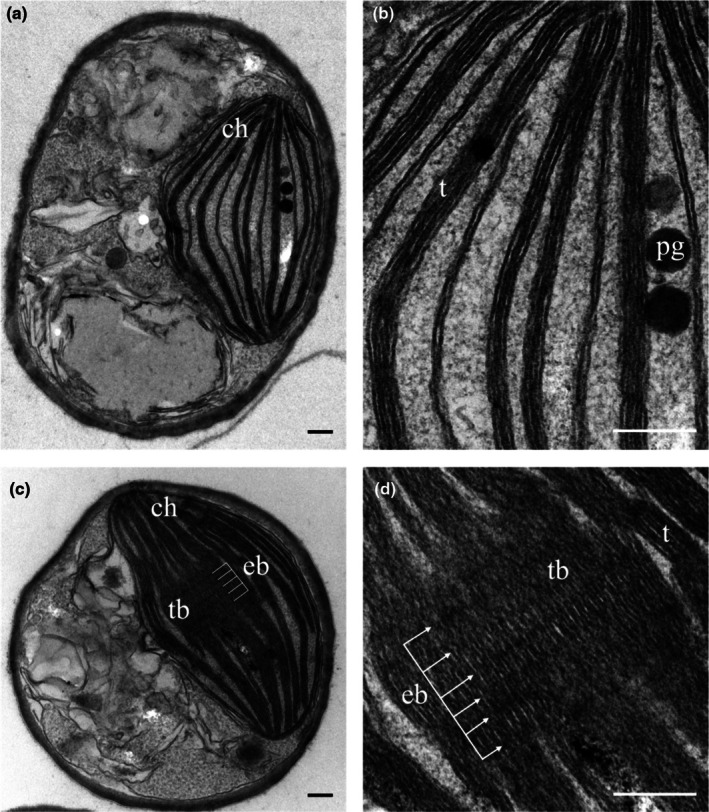
Representative transmission electron microscopy micrographs of SOL‐acclimated (a, b) and FR‐acclimated (c, d) *Nannochloropsis gaditana* cells. Panels b and d show magnifications focusing on the thylakoid membrane organization and thylakoid body in FR. The electron‐dense bands (eb) running perpendicularly to the thylakoids in FR light are indicated with white lines in (c) and arrows in (d). ch, chloroplast; eb, electron‐dense bands; FR, far‐red light; pg, plastoglobules; SOL, solar‐like light; t, thylakoids; TB, thylakoidal bodies. Bars, 200 nm.

**Fig. 6:**
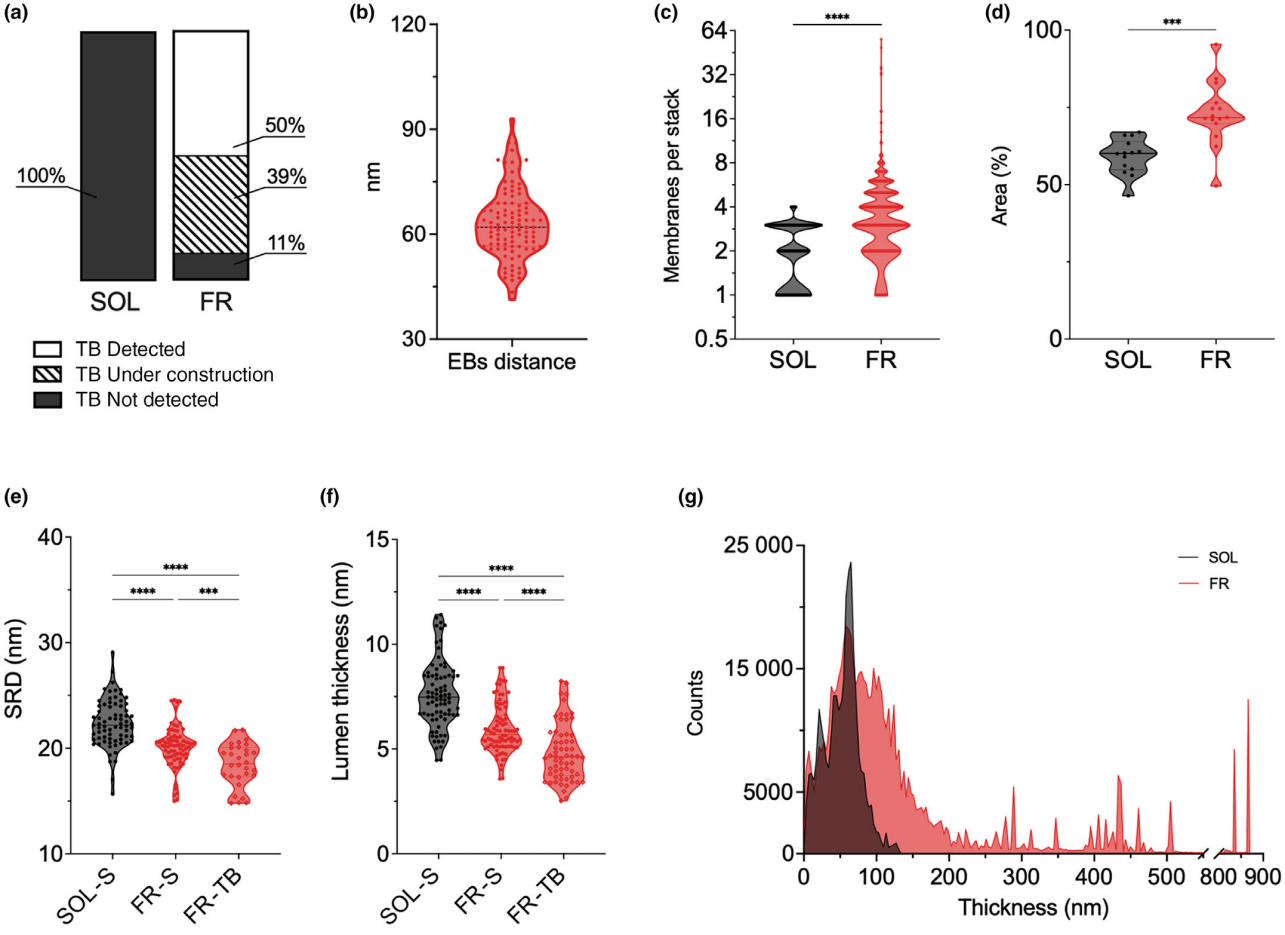
Morphometric analysis of SOL‐ and FR‐acclimated *Nannochloropsis gaditana* cells. (a) Percentage of *TB* detection in SOL‐ and FR‐acclimated imaged cells; (b) distance between electron‐dense bands (EBs) (*n* measures = 100); (c) number of thylakoid membranes per stack (Mann–Whitney test, 150 stacks per condition, *P* < 0.001); (d) percentage of total chloroplast's area occupied by thylakoid membranes (Mann–Whitney test, ≥ 339 cells imaged per condition, *P* < 0.0001); (e) stacking repeat distance (SRD), considering the differences between stacks (SOL‐S and FR‐S) and TB (Mann–Whitney test, ≥ 30 stacks per condition, *P* < 0.001); (f) lumen thickness, considering the differences between stacks (SOL‐S and FR‐S) and TB (Mann–Whitney test, ≥ 62 membranes per condition, *P* < 0.0001); (g) average local thickness in terms of counts, expressing the frequency of each thickness value registered in the stacks. Lines in the violin plots indicate the mean value. FR, far‐red light; SOL, solar‐like light; SRD, stacking repeat distance; TB, thylakoidal bodies.

To investigate the implications of the observed functional alterations of FR‐acclimated cells on the chloroplasts' ultrastructure, these were imaged with transmission electron microscopy. SOL‐acclimated cells presented a short number of small stacks of thylakoid membranes (Fig. 5a,b) as expected in this species (Simionato *et al*., 2013). On the other hand, FR‐acclimated chloroplasts showed more photosynthetic membranes arranged in much larger stacks (Fig. 5c,d), frequently merging in highly regular superstacks, with electron‐dense bands (Fig. 5d, arrows) running perpendicularly to the orientation of thylakoid membranes. To the best of our knowledge, this is the first observation of such thylakoidal structures. We propose to name these newly observed structures as *thylakoidal* *bodies*, TBs. In particular, TBs can be described as an ordered large block of strongly appressed parallel membranes, crossed by perpendicular electron‐dense bands spread across all the superstack (Figs 5c,d, S8). TBs either appeared completely formed (Figs 4c,d, 6a, S3f) or under construction (Figs S3, S8), and they were never detected in SOL‐acclimated cells. In some FR micrographs, it was possible to see small portions of formed TBs accompanied by unordered electron‐dense matter in their proximity and in between adjacent stacks (Fig. S3e). In these cases, TBs were classified as under construction. In formed TBs, the perpendicular electron dense bands were regularly spaced one from the other, at a mean distance of 63 nm (Fig. 6b).

When counted, in FR‐acclimated cells, each thylakoid stack was composed of an average of 5 membranes, with a maximum of 56 membranes per stack, whereas in SOL, stacks showed from a minimum of 1 to a maximum of 4 membranes (Fig. 6c). In FR, the presence of TBs and bigger stacks, resulted in a higher percentage of the chloroplast area occupied by membranes (Fig. 6d). To evaluate the average thickness of the stacks' layers, the stacking repeat distance (SRD) value was calculated as the ratio between the thickness of a stack and the number of membranes that composed it. This value, together with the thickness of the membrane lumen, indicates the stacking level of thylakoid membranes, meaning how packed the membranes are to each other inside the stack. SRD, together with the measured lumen thickness, sensibly changed in the two light conditions. In FR‐acclimated cells, the stacking of the membranes and their thylakoid lumen thickness were significantly lower, especially when considering only TBs (Fig. 6e,f). The acclimation to FR light triggered a tighter membrane system, with stacks composed of a higher number of membranes that were more compressed than what exhibited in SOL. This condition appeared to be more pronounced in TBs, where the registered SRD and lumen thickness were even shorter. The width of thylakoid stacks was considered as well and was evaluated via the average local thickness value. This was calculated through the ‘local thickness’ tool in ImageJ, which results in the distribution of frequencies of the thickness values measured in micrographs expressed as *counts*, meaning the number of times each value of thickness was registered (Fig. 6g). Concurrently with tighter membranes, the width of thylakoid stacks was also found to be increased upon FR acclimation: the stack thickness registered was mainly between 3 nm and 500 nm, with exceptions up to *c*. 850 nm, whereas in SOL light, it was only between 3 nm and 130 nm.

## Discussion

In this work, we demonstrated for the first time that a microalga species, the Eustigmatophyte *Nannochloropsis gaditana*, is capable of long‐term growth under monochromatic 730 nm FR light without a differential rise in absorption beyond 700 nm. According to the absorption and transmission spectra, there is no significant shift in the spectral forms present in the cells and no significant red shift in the Chl*a* absorption.

This observation suggests that the ability of *N. gaditana* to grow in FR light does not depend on the synthesis of red‐shifted antenna complexes or pigments, and it thus must rely on the ability of red‐shifted Chls present in PSI to absorb FR light. In this context, it is interesting to observe that isolated PSI particles from *Nannochloropsis* have been shown to have a significant absorption over 700 nm at 77 K with a tail going over 710 nm and beyond (Alboresi *et al*., 2017). A PSI that is intrinsically more red‐shifted could mark the difference with other organisms that cannot grow in FR light nor have the ability to modify their absorption features in order to harvest longer wavelengths.

This PSI absorption, however, is accompanied by the activation of a specific acclimation to FR light that differs from the two strategies identified so far in microalgae or cyanobacteria for supporting their growth in FR light, involving the synthesis of specific proteins and/or pigments. *N. gaditana* acclimated to FR light, in fact, shows some of the responses typically induced by low light, such as the increase in pigment content per cell coupled with the rise in Chl : car ratio (Fig. 1) and the increase in the content of all photosynthetic complexes, both PSI and PSII (Fig. 2) (Meneghesso *et al*., 2016). The strong increase in pigment concentration clearly contributes to a higher overall capacity for light harvesting.

Upon acclimation to FR light, however, there are also responses that are different from the ones observed in low light. One is the change in the PSII : PSI ratio, which is much higher in FR than in low light. As FR light is preferentially absorbed by PSI (Gobets & Van Grondelle, 2001), the adjusted content and stoichiometry of photosystems maximize the harvesting of available light by PSII and compensate for the overexcitation of PSI by FR light (Chow *et al*., 1990). As it suboptimally harvests FR light, PSII becomes one of the most limiting factors for photosynthetic efficiency in this light condition. A similar change in PSs abundance was also observed in plants exposed to FR light (Hu *et al*., 2021; Leschevin *et al*., 2024).

A second specific FR response is the reduction of PSII antenna size (Fig. 2), while the opposite was observed upon low‐light acclimation on *N. gaditana*, which showed larger antenna size (Meneghesso *et al*., 2016). As almost all the residual visible light in FR conditions is in the red waveband (1.69 μmol of photons m^−2^ s^−1^), it is interesting to point out that the acclimation response observed in this work is also different from the one displayed by *N. gaditana* cells acclimated to red light, where Chl content decreases and the Chl to carotenoid ratio rises. This again supports the fact that the acclimation we describe is a specific response to FR light (Kim *et al*., 2014).

The reduction in antenna size in FR light is consistent with the observation that no red‐shifted antennas are accumulated in *N. gaditana*: the little absorption in the FR of PSII is likely associated with PSII core and thus reduces the need for a larger antenna and stimulates accumulation of core complexes (Sirohiwal & Pantazis, 2022). Upon FR acclimation, electron transport activities are also affected. Results showed a higher effective PSII efficiency in FR‐acclimated cells in the dark, but the efficiency decreases much faster as soon as cells are illuminated (Fig. 3). This suggests that cells are not able to sustain a strong photosynthetic activity in visible light, and electron transport is easily saturated. This can be explained by considering that PSII/PSI is unbalanced, and in these cells, PSI activity will rapidly become limiting under white light because of its lower accumulation, as shown by the qL values that indicate PQ overreduction with very dim light intensities.

### Reorganization of thylakoid structure contributes to FR acclimation

Another specific response to FR acclimation is the modification of chloroplasts' ultrastructure with the formation of very large thylakoid stacks with a peculiar organization. In FR light, chloroplasts were bigger and occupied a larger fraction of the cell, hosting a much larger number of thylakoid membranes than in SOL.

An increase in thylakoid membranes is typical of low light acclimation (Meneghesso *et al*., 2016), but upon FR light acclimation, this tendency is exacerbated, with superstacks that counted up to 56 membranes, reaching a size of 500 nm. SOL‐acclimated stacks were instead composed of a maximum of four membranes. Thylakoids acclimated to FR light were also more tightly packed, with a reduction in lumen thickness compared with SOL‐acclimated chloroplasts (Figs 5, 6, S8). Moreover, in FR‐acclimated chloroplasts, the large stacks often converged into a peculiar, highly ordered structure composed of several stacked thylakoid membranes, which we proposed to name *thylakoidal* *bodies,* TBs. TBs were also characterized by electron‐dense bands running orthogonal to the membranes, regularly spaced from each other at a mean distance of 63 ± 9 nm. It is interesting to observe that these dense bands on TEM images show some similarity with *Z* bands observed in sarcomeres, which could open up the hypothesis that they could have a similar function in anchoring thylakoidal membranes (Luther, 2009). TBs were detected in 50% of the FR‐acclimated cells as completely formed, whereas in 39%, they were identified as under assembly. Most importantly, TBs were never detected in SOL‐acclimated cells.

Interestingly, a highly regular organization of thylakoid membrane was found in the iridoplasts (epidermal chloroplasts) of *Begonia* plants living in the tropical forest understory, where they are exposed to low light enriched in FR (Endler, 1993; Gould & Lee, 1996). These structures were found to increase the capture of light filtering from the upper canopy and to enhance quantum yield up to 10% under low light conditions (Jacobs *et al*., 2016). With a similar role are the bizonoplasts, chloroplasts found in species of the vascular plant family of *Selaginellaceae*, adapted to low‐light spectra enriched in FR (Liu *et al*., 2020). These peculiar chloroplasts have a dimorphic structure, where the upper zone has no grana and is made up of *c*. 11 parallel thylakoid groups, each containing three to five stacked thylakoids, regularly spaced by the stroma, while the lower zone is typical of higher plants (with grana and stroma thylakoids) (Liu *et al*., 2020). In both cases, the specific distance in between membrane layers is thought to interfere with light waves, enhancing the absorption of FR photons and optimizing light harvesting in shaded environments (Jacobs *et al*., 2016; Liu *et al*., 2020).

These ordered structures have different sizes and membrane distances than the ones observed here; furthermore, they can generate a spectroscopic signature (Pao *et al*., 2018), which is not the case for TBs. The functional explanation of the TBs observed here must thus rely on other phenomena. Capretti *et al*. (2019) demonstrated that grana thylakoids can generate nanophotonic effects, including directional scattering, light trapping and harvesting enhancement, depending on their nanoscale morphology. Following these hypotheses, one possibility is that TBs could contribute to light scattering. Light scattering is in fact usually modeled as a sum of different contributions from rays refracted by interfaces with different refraction indices (Baránková *et al*., 2020) when dealing with scattering particles sized larger than several wavelengths. Scattering for small particles is described by the Rayleigh equations, and it is proportional to 1/λ^4^ (Kleinman & Senior, 1986); therefore, it is not very effective for longer wavelengths. If the particles are larger, with a size comparable to the light wavelength, instead, scattering is better described by the Mie approximation, where its intensity does not depend on the λ (Wriedt, 2012). This suggests that by increasing the size of the scattering particles and reaching dimensions approximately close to λ the scattering would increase, particularly for the longer wavelengths that were less affected before. If this hypothesis is correct, the TBs, by having sizes of up to 500 nm, will increase the scattering of FR light within the cell, thus increasing the chances that the small absorption tail, associated with PSI red‐shifted forms, would be sufficient to provide enough light absorption to support growth.

This could be an alternative response of the thylakoid organization to succeed under FR light, respecting the one adopted by understory shade‐adapted plants. This strategy would be better suited for a free‐living unicellular organism like *Nannochloropsis*. Structures like iridoplasts, in fact, are effective because in the leaf tissue, the 3D structures can be oriented with the light that is always coming from the same direction. For a free‐living unicellular coccoid alga, the light direction is continuously changing and thus these oriented structures would be much less effective. Exploiting a phenomenon like scattering that does not require orientation would enable to maximize the chances of light absorption without any directionality.

The presence of a high number of membranes or superstacks is not a unique strategy of this species upon shading or FR irradiation. Superstacks have also been reported in *Phaeodactylum tricornutum*, another unicellular alga, even if this species concurrently accumulates red‐shifted antennae upon FR exposure (Bína *et al*., 2016; Herbstová *et al*., 2017). Interestingly, both *P. tricornutum* and *N. gaditana* are found freely in seawater, suggesting that this structural trait could be more advantageous for a free‐living cell where the light has no specific directionality; increasing scattering of long wavelengths would be effective in increasing light absorption efficiency.

The hypothesis of a relevant role of superstacks under FR light is also supported by their observation in plants exposed to FR light or shaded FR‐enriched spectra (Hu *et al*., 2021; Colpo *et al*., 2023; Leschevin *et al*., 2024) and in photosynthetic stems where chloroplasts receive very low visible FR‐enriched light (Natale *et al*., 2023).

In conclusion, the structural reorganization presented here reveals a novel mechanism of acclimation to increase the absorption of FR light, highlighting the biodiversity of this response and opening up the possibility that FR acclimation capacity may be more widespread than expected, even in microalgal species that do not display red shifts in absorbance.

## Competing interests

None declared.

## Author contributions

EL contributed to the conceptualization, methodology, investigation, formal analysis and data curation, participated in writing the original draft, and reviewing and editing. MB contributed to the conceptualization and participated in writing the original draft and reviewing and editing the manuscript. MS performed formal analysis and data curation, and assisted with manuscript review and editing. BB performed formal analysis and data curation, and assisted with manuscript review and editing. LC contributed to the methodology and the review and editing of the manuscript. GP contributed to the conceptualization, methodology, resources, and funding acquisition, and was involved in reviewing and editing the manuscript, TM contributed to the conceptualization and supervision, participated in writing the original draft, and reviewed and edited the manuscript. NLR contributed to the conceptualization, supervision, methodology, data curation, resources, and funding acquisition, and was involved in writing the original draft and reviewing and editing the manuscript.

## Disclaimer

The New Phytologist Foundation remains neutral with regard to jurisdictional claims in maps and in any institutional affiliations.

## Supporting information


**Dataset S1** Datasheet for Figs 1‐4 and Fig. 6.

**Fig. S1** Light spectra employed in the work.
**Fig. S2** Experimental setup for the reflectivity measurements.
**Fig. S3** Representative images as references for the detection of *thylakoidal bodies*.
**Fig. S4** Pipeline of image processing for the local thickness analysis.
**Fig. S5** Appearance of acclimated cultures.
**Fig. S6** Western blot analysis.
**Fig. S7** P700 oxidation and reduction kinetics.
**Fig. S8** Representative micrographs of cells.
**Table S1** Summary of the far‐red light using algae and relative strategies.
**Table S2** Repartition of light in the spectra used depending on the waveband.
Please note: Wiley is not responsible for the content or functionality of any Supporting Information supplied by the authors. Any queries (other than missing material) should be directed to the *New Phytologist* Central Office.

## Data Availability

The data that support the findings of this study are included as part of the manuscript or as Supporting Information in Dataset S1 and in Figs S1–S8.

## References

[nph70786-bib-0001] Alboresi A , Le Quiniou C , Yadav SKN , Scholz M , Meneghesso A , Gerotto C , Simionato D , Hippler M , Boekema EJ , Croce R *et al*. 2017. Conservation of core complex subunits shaped the structure and function of photosystem I in the secondary endosymbiont alga *Nannochloropsis gaditana* . New Phytologist 213: 714–726. 27620972 10.1111/nph.14156 PMC5216901

[nph70786-bib-0002] Amesz J , Duysens LNM , Brandt DC . 1961. Methods for measuring and correcting the absorption spectrum of scattering suspensions. Journal of Theoretical Biology 1: 59–74. 13682995 10.1016/0022-5193(61)90026-1

[nph70786-bib-0003] Antonaru LA , Rad‐Menéndez C , Mbedi S , Sparmann S , Pope M , Oliver T , Wu S , Green DH , Gugger M , Nürnberg DJ . 2025. Evolution of far‐red light photoacclimation in cyanobacteria. Current Biology 35: 2539–2553. 40367945 10.1016/j.cub.2025.04.038

[nph70786-bib-0004] Arshad R , Saccon F , Bag P , Biswas A , Calvaruso C , Bhatti AF , Grebe S , Mascoli V , Mahbub M , Muzzopappa F *et al*. 2022. A kaleidoscope of photosynthetic antenna proteins and their emerging roles. Plant Physiology 189: 1204–1219. 35512089 10.1093/plphys/kiac175 PMC9237682

[nph70786-bib-0005] Bailleul B , Cardol P , Breyton C , Finazzi G . 2010. Electrochromism: a useful probe to study algal photosynthesis. Photosynthesis Research 106: 179–189. 20632109 10.1007/s11120-010-9579-z

[nph70786-bib-0006] Baránková B , Lazár D , Nauš J , Solovchenko A , Gorelova O , Baulina O , Huber G , Nedbal L . 2020. Light absorption and scattering by high light‐tolerant, fast‐growing *Chlorella vulgaris* IPPAS C‐1 cells. Algal Research 49: 101881.

[nph70786-bib-0007] Battistuzzi M , Cocola L , Liistro E , Claudi R , Poletto L , La Rocca N . 2023. Growth and photosynthetic efficiency of microalgae and plants with different levels of complexity exposed to a simulated M‐Dwarf starlight. Life 13: 81641. 10.3390/life13081641 PMC10455698 37629498

[nph70786-bib-0008] Battistuzzi M , Cocola L , Salasnich B , Erculiani MS , Alei E , Morosinotto T , Claudi R , Poletto L , La Rocca N . 2020. A new remote sensing‐based system for the monitoring and analysis of growth and gas exchange rates of photosynthetic microorganisms under simulated non‐terrestrial conditions. Frontiers in Plant Science 11: 182. 32210991 10.3389/fpls.2020.00182 PMC7066451

[nph70786-bib-0009] Bilger W , Björkman O . 1990. Role of the xanthophyll cycle in photoprotection elucidated by measurements of light‐induced absorbance changes, fluorescence and photosynthesis in leaves of *Hedera canariensis* . Photosynthesis Research 25: 173–185. 24420348 10.1007/BF00033159

[nph70786-bib-0010] Bína D , Gardian Z , Herbstová M , Kotabová E , Koník P , Litvín R , Prášil O , Tichý J , Vácha F . 2014. Novel type of red‐shifted chlorophyll a antenna complex from *Chromera velia*: II. Biochemistry and spectroscopy. Biochimica et Biophysica Acta ‐ Bioenergetics 1837: 802–810. 10.1016/j.bbabio.2014.01.011 24486443

[nph70786-bib-0011] Bína D , Herbstová M , Gardian Z , Vácha F , Litvín R . 2016. Novel structural aspect of the diatom thylakoid membrane: lateral segregation of photosystem I under red‐enhanced illumination. Scientific Reports 6: 25583. 27149693 10.1038/srep25583 PMC4857733

[nph70786-bib-0012] Bryant DA , Canniffe DP . 2018. How nature designs light‐harvesting antenna systems: Design principles and functional realization in chlorophototrophic prokaryotes. Journal of Physics B: Atomic, Molecular and Optical Physics 51: 33001.

[nph70786-bib-0013] Capretti A , Ringsmuth AK , Van Velzen JF , Rosnik A , Croce R , Gregorkiewicz T . 2019. Nanophotonics of higher‐plant photosynthetic membranes. Light: Science & Applications 8: 5. 10.1038/s41377-018-0116-8 PMC6325066 30651980

[nph70786-bib-0014] Chen M . 2014. Chlorophyll modifications and their spectral extension in oxygenic photosynthesis. Annual Review of Biochemistry 83: 317–340. 10.1146/annurev-biochem-072711-162943 24635479

[nph70786-bib-0015] Chen M , Li Y , Birch D , Willows RD . 2012. A cyanobacterium that contains chlorophyll *f* – a red‐absorbing photopigment. FEBS Letters 586: 3249–3254. 22796191 10.1016/j.febslet.2012.06.045

[nph70786-bib-0016] Chow WS , Melis A , Anderson JM . 1990. Adjustments of photosystem stoichiometry in chloroplasts improve the quantum efficiency of photosynthesis. Proceedings of the National Academy of Sciences, USA 87: 7502–7506. 10.1073/pnas.87.19.7502 PMC54775 11607105

[nph70786-bib-0017] Colpo A , Molinari A , Boldrini P , Živčak M , Brestič M , Demaria S , Baldisserotto C , Pancaldi S , Ferroni L . 2023. Thylakoid membrane appression in the giant chloroplast of *Selaginella martensii* spring: a lycophyte challenges grana paradigms in shade‐adapted species. Plant Science 336: 111833. 37595894 10.1016/j.plantsci.2023.111833

[nph70786-bib-0018] Cruz JA , Sacksteder CA , Kanazawa A , Kramer DM . 2001. Contribution of electric field (Δψ) to steady‐state transthylakoid proton motive force (pmf) *in vitro* and *in vivo*. Control of pmf parsing into Δψ and ΔpH by ionic strength. Biochemistry 40: 1226–1237. 11170448 10.1021/bi0018741

[nph70786-bib-0019] Elias E , Oliver TJ , Croce R . 2024. Oxygenic photosynthesis in far‐red light: strategies and mechanisms. Annual Review of Physical Chemistry 75: 231–256. 10.1146/annurev-physchem-090722-125847 38382567

[nph70786-bib-0020] Endler JA . 1993. The color of light in forests and its implications. Ecological Monographs 63: 1–27.

[nph70786-bib-0021] Fawley MW , Jameson I , Fawley KP . 2015. The phylogeny of the genus *Nannochloropsis* (Monodopsidaceae, Eustigmatophyceae), with descriptions of *N. australis* sp. Nov. and *Microchloropsis* gen. Nov. Phycologia 54: 545–552.

[nph70786-bib-0022] Gan F , Bryant DA . 2015. Adaptive and acclimative responses of cyanobacteria to far‐red light. Environmental Microbiology 17: 3450–3465. 26234306 10.1111/1462-2920.12992

[nph70786-bib-0023] Gan F , Shen G , Bryant DA . 2015. Occurrence of far‐red light photoacclimation (FaRLiP) in diverse cyanobacteria. Life 5: 10004. 10.3390/life5010004 PMC4390838 25551681

[nph70786-bib-0024] Gan F , Zhang S , Rockwell NC , Martin SS , Lagarias JC , Bryant DA . 2014. Extensive remodeling of a cyanobacterial photosynthetic apparatus in far‐red light. Science 345: 1312–1317. 25214622 10.1126/science.1256963

[nph70786-bib-0025] Genty B , Briantais J‐M , Baker NR . 1989. The relationship between the quantum yield of photosynthetic electron transport and quenching of chlorophyll fluorescence. Biochimica et Biophysica Acta (BBA) ‐ General Subjects 990: 87–92.

[nph70786-bib-0026] Gobets B , Van Grondelle R . 2001. Energy transfer and trapping in photosystem I. Biochimica et Biophysica Acta ‐ Bioenergetics 1507: 80–99. 10.1016/s0005-2728(01)00203-1 11687209

[nph70786-bib-0027] Gould KS , Lee DW . 1996. Physical and ultrastructural basis of blue leaf iridescence in four Malaysian understory plants. American Journal of Botany 83: 45–50.

[nph70786-bib-0028] Guillard RR , Ryther JH . 1962. Studies of marine planktonic diatoms. I. Cyclotella nana Hustedt, and Detonula confervacea (cleve) Gran. Canadian Journal of Microbiology 8: 229–239. 13902807 10.1139/m62-029

[nph70786-bib-0029] Herbstová M , Bína D , Kaňa R , Vácha F , Litvín R . 2017. Red‐light phenotype in a marine diatom involves a specialized oligomeric red‐shifted antenna and altered cell morphology. Scientific Reports 7: 11976. 28931902 10.1038/s41598-017-12247-0 PMC5607283

[nph70786-bib-0030] Herbstová M , Bína D , Koník P , Gardian Z , Vácha F , Litvín R . 2015. Molecular basis of chromatic adaptation in pennate diatom *Phaeodactylum tricornutum* . Biochimica et Biophysica Acta 1847: 534–543. 25748970 10.1016/j.bbabio.2015.02.016

[nph70786-bib-0031] Hu C , Nawrocki WJ , Croce R . 2021. Long‐term adaptation of *Arabidopsis thaliana* to far‐red light. Plant, Cell & Environment 44: 3002–3014. 10.1111/pce.14032 PMC8453498 33599977

[nph70786-bib-0032] Hulatt CJ , Wijffels RH , Bolla S , Kiron V . 2017. Production of fatty acids and protein by *Nannochloropsis* in flat‐plate photobioreactors. PLoS ONE 12: e0170440. 28103296 10.1371/journal.pone.0170440 PMC5245880

[nph70786-bib-0033] Jacobs M , Lopez‐Garcia M , Phrathep O‐P , Lawson T , Oulton R , Whitney HM . 2016. Photonic multilayer structure of Begonia chloroplasts enhances photosynthetic efficiency. Nature Plants 2: 16162. 27775728 10.1038/nplants.2016.162

[nph70786-bib-0034] Kim CW , Sung M‐G , Nam K , Moon M , Kwon J‐H , Yang J‐W . 2014. Effect of monochromatic illumination on lipid accumulation of *Nannochloropsis gaditana* under continuous cultivation. Bioresource Technology 159: 30–35. 24632438 10.1016/j.biortech.2014.02.024

[nph70786-bib-0035] Kleinman RE , Senior TBA . 1986. Rayleigh scattering. In: Mechanics and mathematical methods–series of handbooks, vol. 2. London, UK: Elsevier, 1–70. doi: 10.1016/B978-0-444-87726-0.50006-9.

[nph70786-bib-0036] Koehne B , Elli G , Jennings RC , Wilhelm C , Trissl H‐W . 1999. Spectroscopic and molecular characterization of a long wavelength absorbing antenna of *Ostreobium* sp. Biochimica et Biophysica Acta ‐ Bioenergetics 1412: 94–107. 10.1016/s0005-2728(99)00061-4 10393253

[nph70786-bib-0037] Kosugi M , Kawasaki M , Shibata Y , Hara K , Takaichi S , Moriya T , Adachi N , Kamei Y , Kashino Y , Kudoh S *et al*. 2023. Uphill energy transfer mechanism for photosynthesis in an Antarctic alga. Nature Communications 14: 730. 10.1038/s41467-023-36245-1 PMC9931709 36792917

[nph70786-bib-0038] Kosugi M , Ozawa S‐I , Takahashi Y , Kamei Y , Itoh S , Kudoh S , Kashino Y , Koike H . 2020. Red‐shifted chlorophyll a bands allow uphill energy transfer to photosystem II reaction centers in an aerial green alga, *Prasiola crispa*, harvested in Antarctica. Biochimica et Biophysica Acta ‐ Bioenergetics 1861: 148139. 31825812 10.1016/j.bbabio.2019.148139

[nph70786-bib-0039] Kotabová E , Jarešová J , Kaňa R , Sobotka R , Bína D , Prášil O . 2014. Novel type of red‐shifted chlorophyll a antenna complex from *Chromera velia*. I. Physiological relevance and functional connection to photosystems. Biochimica et Biophysica Acta ‐ Bioenergetics 1837: 734–743. 10.1016/j.bbabio.2014.01.012 24480388

[nph70786-bib-0040] Kourra N , Warnett JM , Attridge A , Dibling G , McLoughlin J , Muirhead‐Allwood S , King R , Williams MA . 2018. Computed tomography metrological examination of additive manufactured acetabular hip prosthesis cups. Additive Manufacturing 22: 146–152.

[nph70786-bib-0041] Kramer DM , Johnson G , Kiirats O , Edwards GE . 2004. New fluorescence parameters for the determination of Q_A_ redox state and excitation energy fluxes. Photosynthesis Research 79: 209–218. 16228395 10.1023/B:PRES.0000015391.99477.0d

[nph70786-bib-0042] La Rocca N , Moro I , Battistuzzi M , Rascio N . 2024. Cyanobacteria and microalgae responses to different light regimes and CO_2_ availability. In: Pessarakli M , ed. Handbook of photosynthesis, 4^th^ edn. Boca Raton, FL, USA: CRC Press, 296–350. doi: 10.1201/b22922-21.

[nph70786-bib-0043] Lamb JJ , Røkke G , Hohmann‐Marriott MF . 2018. Chlorophyll fluorescence emission spectroscopy of oxygenic organisms at 77 K. Photosynthetica 56: 105–124.

[nph70786-bib-0044] Lazar D , Stirbet A , Björn LO , Govindjee G . 2022. Light quality, oxygenic photosynthesis and more. Photosynthetica 60: 25–58. 39648998 10.32615/ps.2021.055 PMC11559484

[nph70786-bib-0045] Leschevin M , Ksas B , Baltenweck R , Hugueney P , Caffarri S , Havaux M . 2024. Photosystem rearrangements, photosynthetic efficiency, and plant growth in far red‐enriched light. The Plant Journal 120: 2536–2552. 39506623 10.1111/tpj.17127 PMC11658187

[nph70786-bib-0046] Li M , Mukhopadhyay R , Svoboda V , Oung HMO , Mullendore DL , Kirchhoff H . 2020. Measuring the dynamic response of the thylakoid architecture in plant leaves by electron microscopy. Plant Direct 4: e00280. 33195966 10.1002/pld3.280 PMC7644818

[nph70786-bib-0047] Litvín R , Bína D , Herbstová M , Pazderník M , Kotabová E , Gardian Z , Trtílek M , Prášil O , Vácha F . 2019. Red‐shifted light‐harvesting system of freshwater eukaryotic alga *Trachydiscus minutus* (Eustigmatophyta, Stramenopila). Photosynthesis Research 142: 137–151. 31375979 10.1007/s11120-019-00662-5

[nph70786-bib-0048] Liu J , Song Y , Qiu W . 2017. Oleaginous microalgae *Nannochloropsis* as a new model for biofuel production: review & analysis. Renewable and Sustainable Energy Reviews 72: 154–162.

[nph70786-bib-0049] Liu J‐W , Li S‐F , Wu C‐T , Valdespino IA , Ho J‐F , Wu Y‐H , Chang H‐M , Guu T‐Y , Kao M‐F , Chesson C *et al*. 2020. Gigantic chloroplasts, including bizonoplasts, are common in shade‐adapted species of the ancient vascular plant family Selaginellaceae. American Journal of Botany 107: 562–576. 32227348 10.1002/ajb2.1455

[nph70786-bib-0050] Loughlin P , Lin Y , Chen M . 2013. Chlorophyll d and *Acaryochloris marina*: current status. Photosynthesis Research 116: 277. 23615924 10.1007/s11120-013-9829-y

[nph70786-bib-0051] Lubián LM , Montero O , Moreno‐Garrido I , Huertas IE , Sobrino C , Valle MG , Pares G . 2000. *Nannochloropsis* (Eustigmatophyceae) as source of commercially valuable pigments. Journal of Applied Phycology 12: 249–255.

[nph70786-bib-0052] Luther PK . 2009. The vertebrate muscle Z‐disc: sarcomere anchor for structure and signalling. Journal of Muscle Research and Cell Motility 30: 171–185. 19830582 10.1007/s10974-009-9189-6 PMC2799012

[nph70786-bib-0053] Lyu H , Lazár D . 2022. Analyzing the effect of ion binding to the membrane‐surface on regulating the light‐induced transthylakoid electric potential (ΔΨm). Frontiers in Plant Science 13: 675. 10.3389/fpls.2022.945675 PMC9366520 35968094

[nph70786-bib-0054] Ma X‐N , Chen T‐P , Yang B , Liu J , Chen F . 2016. Lipid production from *Nannochloropsis* . Marine Drugs 14: 61. 27023568 10.3390/md14040061 PMC4849066

[nph70786-bib-0055] Martín‐de León J , Bernardo V , Rodríguez‐Pérez M . 2016. Low density nanocellular polymers based on PMMA produced by gas dissolution foaming: fabrication and cellular structure characterization. Polymers 8: 265. 30974541 10.3390/polym8070265 PMC6432238

[nph70786-bib-0056] Mazur R , Mostowska A , Kowalewska Ł . 2021. How to measure grana – ultrastructural features of thylakoid membranes of plant chloroplasts. Frontiers in Plant Science 12: 756009. 34691132 10.3389/fpls.2021.756009 PMC8527009

[nph70786-bib-0057] Meneghesso A , Simionato D , Gerotto C , La Rocca N , Finazzi G , Morosinotto T . 2016. Photoacclimation of photosynthesis in the eustigmatophycean *Nannochloropsis gaditana* . Photosynthesis Research 129: 291–305. 27448115 10.1007/s11120-016-0297-z

[nph70786-bib-0058] Miyashita H , Ikemoto H , Kurano N , Adachi K , Chihara M , Miyachi S . 1996. Chlorophyll d as a major pigment. Nature 383: 402.

[nph70786-bib-0059] Morosinotto T , Breton J , Bassi R , Croce R . 2003. The nature of a chlorophyll ligand in Lhca proteins determines the far red fluorescence emission typical of photosystem I. Journal of Biological Chemistry 278: 49223–49229. 14504274 10.1074/jbc.M309203200

[nph70786-bib-0060] Natale S , La Rocca N , Battistuzzi M , Morosinotto T , Nardini A , Alboresi A . 2023. Structure and function of bark and wood chloroplasts in a drought‐tolerant tree (*Fraxinus ornus* L.). Tree Physiology 43: 893–908. 36738252 10.1093/treephys/tpad013

[nph70786-bib-0061] Onami C , Nishibori Y , Suda S , Kamikawa R , Miyashita H . 2025. Far‐red light acclimation in *Phaeophila dendroides* (Ulvales, Ulvophyceae) isolated from a coral skeleton. Phycological Research 73: 27–34.

[nph70786-bib-0062] Oxborough K , Baker NR . 1997. Resolving chlorophyll a fluorescence images of photosynthetic efficiency into photochemical and non‐photochemical components – calculation of qP and *F* _v_−/*F* _m_−; without measuring *F* _0_−. Photosynthesis Research 54: 135–142.

[nph70786-bib-0063] Pao S‐H , Tsai P‐Y , Peng C‐I , Chen P‐J , Tsai C‐C , Yang E‐C , Shih M‐C , Chen J , Yang J‐Y , Chesson P *et al*. 2018. Lamelloplasts and minichloroplasts in Begoniaceae: iridescence and photosynthetic functioning. Journal of Plant Research 131: 655–670. 29500749 10.1007/s10265-018-1020-2

[nph70786-bib-0064] Perin G , Bellan A , Segalla A , Meneghesso A , Alboresi A , Morosinotto T . 2015. Generation of random mutants to improve light‐use efficiency of *Nannochloropsis gaditana* cultures for biofuel production. Biotechnology for Biofuels 8: 161. 26413160 10.1186/s13068-015-0337-5 PMC4583171

[nph70786-bib-0065] Perin G , Simionato D , Bellan A , Carone M , Occhipinti A , Maffei ME , Morosinotto T . 2017. Cultivation in industrially relevant conditions has a strong influence on biological properties and performances of *Nannochloropsis gaditana* genetically modified strains. Algal Research 28: 88–99.

[nph70786-bib-0066] Raven J . 2009. Contributions of anoxygenic and oxygenic phototrophy and chemolithotrophy to carbon and oxygen fluxes in aquatic environments. Aquatic Microbial Ecology 56: 177–192.

[nph70786-bib-0067] Shibata K . 1959. Spectrophotometry of translucent biological materials—opal glass transmission method. In: Glick D , ed. Methods of biochemical analysis, 1^st^ edn. Hoboken, NJ, USA: Wiley, 77–109. doi: 10.1002/9780470110232.ch3.

[nph70786-bib-0068] Simionato D , Block MA , La Rocca N , Jouhet J , Maréchal E , Finazzi G , Morosinotto T . 2013. The response of *Nannochloropsis gaditana* to nitrogen starvation includes *de novo* biosynthesis of triacylglycerols, a decrease of chloroplast galactolipids, and reorganization of the photosynthetic apparatus. Eukaryotic Cell 12: 665–676. 23457191 10.1128/EC.00363-12 PMC3647774

[nph70786-bib-0069] Simionato D , Sforza E , Corteggiani Carpinelli E , Bertucco A , Giacometti GM , Morosinotto T . 2011. Acclimation of *Nannochloropsis gaditana* to different illumination regimes: effects on lipids accumulation. Bioresource Technology 102: 6026–6032. 21429740 10.1016/j.biortech.2011.02.100

[nph70786-bib-0070] Sirohiwal A , Pantazis DA . 2022. The electronic origin of far‐red‐light‐driven oxygenic photosynthesis. Angewandte Chemie International Edition 61: e202200356. 35142017 10.1002/anie.202200356 PMC9304563

[nph70786-bib-0071] Sukenik A , Livne A , Neori A , Yacobi YZ , Katcoff D . 1992. Purification and characterization of a light‐harvesting chlorophyll‐protein complex from the marine Eustigmatophyte *Nannochloropsis* sp. Plant and Cell Physiology 20: 8354.

[nph70786-bib-0072] Wang F , Nishibori Y , Kitazaki S , Kamikawa R , Miyashita H . 2025. A new type of photoacclimation to far‐red light found in a newly isolated *Neochloris* sp. (Chlorophyceae, Chlorophyta) from Lake Biwa, Japan. Phycological Research 73: 148–156.

[nph70786-bib-0073] Wellburn AR . 1994. The spectral determination of Chlorophylls a and b, as well as total carotenoids, using various solvents with spectrophotometers of different resolution. Journal of Plant Physiology 144: 307–313.

[nph70786-bib-0074] Wilhelm C , Jakob T . 2006. Uphill energy transfer from long‐wavelength absorbing chlorophylls to PS II in *Ostreobium* sp. Is functional in carbon assimilation. Photosynthesis Research 87: 323–329. 16416051 10.1007/s11120-005-9002-3

[nph70786-bib-0075] Witt HT . 1979. Energy conversion in the functional membrane of photosynthesis. Analysis by light pulse and electric pulse methods. Biochimica et Biophysica Acta (BBA) – Reviews on Bioenergetics 505: 355–427. 10.1016/0304-4173(79)90008-9 35227

[nph70786-bib-0076] Wolf BM , Blankenship RE . 2019. Far‐red light acclimation in diverse oxygenic photosynthetic organisms. Photosynthesis Research 142: 349–359. 31222688 10.1007/s11120-019-00653-6

[nph70786-bib-0077] Wolf BM , Niedzwiedzki DM , Magdaong NCM , Roth R , Goodenough U , Blankenship RE . 2018. Characterization of a newly isolated freshwater *Eustigmatophyte alga* capable of utilizing far‐red light as its sole light source. Photosynthesis Research 135: 177–189. 28547584 10.1007/s11120-017-0401-z

[nph70786-bib-0078] Wriedt T . 2012. Mie theory: a review. In: Hergert W , Wriedt T , eds. The mie theory: basics and applications. New York, NY, USA: Springer, 53–71. doi: 10.1007/978-3-642-28738-1_2.

[nph70786-bib-0079] Zampieri RM , Bizzotto E , Campanaro S , Caldara F , Bellucci M , La Rocca N . 2025. Kovacikia euganea sp. Nov. (Leptolyngbyaceae, Cyanobacteria), a new chlorophyll f producing cyanobacterium from the Euganean Thermal District (Italy). Frontiers in Microbiology 16: 5008. 10.3389/fmicb.2025.1545008 PMC11931122 40130236

[nph70786-bib-0080] Zhao C , Gan F , Shen G , Bryant DA . 2015. RfpA, RfB, and RfC are the master control elements of far‐red light photoacclimation (FaRLiP). Frontiers in Microbiology 6: 1303. 26635768 10.3389/fmicb.2015.01303 PMC4658448

