# Supplementary material for: Thylakoids reorganization enables driving photosynthesis under far‐red light in the microalga Nannochloropsis gaditana
Source: New Phytol. 2025 Dec 3;249(4):1785–99. doi: 10.1111/nph.70786 (PMC12825400; doi:10.1111/nph.70786)

***New Phytologist* Supporting Information**

Article title: Thylakoids reorganization enables driving photosynthesis under far-red light in the microalga *Nannochloropsis gaditana*

Authors: Elisabetta Liistro, Mariano Battistuzzi, Mattia Storti, Beatrice Boccia, Lorenzo Cocola, Giorgio Perin, Tomas Morosinotto, Nicoletta La Rocca

Article acceptance date: 6 November 2025

The following Supporting Information is available for this article:

Table S1 Algal species recognized to operate a far-red light acclimation. n.a.: data not available at the time of writing; FR: far-red. Pc-frLHC: *Prasiola crispa* far-red absorbing light-harvesting complex; PSII: photosystem II; SAR: supergroup including Stramenopiles, Alveolates, Rhizarians; Red-CLH: Red-Chromera light harvesting; rVCP: red-adapted light harvesting complexes.

Organism	Lineage	Habitat	Origin of red-shift	Growth light conditions	References
Prasiola crispa	Green lineage	Aerial(soil), Antarctic, forms thalli, lower cells receive FR-enriched light	Red shifted antenna complexes: Pc-frLHC. Pc-frLHC binds long-wavelength chlorophylls which excites PSII. Pc-frLHC have ring-shaped structure, with chlophyll pentamers, increase probability if uphill energy transfer.	Thalli already acclimated were collected from the environment	(Kosugi et al., 2020, 2023)
***Ostreobium* sp.**	Green lineage	Marine, endozoic (inside corals, shells, under layers of dinoflagellates), extremely low light conditions	Upregulation of Lhca1, which binds long-wavelength chlorophylls. Lhca1 oligomers transfer energy uphill to PSII.	White light with λ < 695 nm cut off filter	(Koehne et al., 1999; Wilhelm & Jakob, 2006)
***Neochloris* sp. Biwa 5-2**	Green lineage	Freshwater, lakeshore	n.a.	Monochromatic FR light at 730 nm, with about 3 µmol of photons m^{-2} s^{-1} < 700 nm	(Wang et al., 2025)
***Phaeophila dendroides* Sa-1**	Green lineage	Marine, coastal waters, endozoic (inside corals)	n.a.	Monochromatic FR light at 730 nm, with about 3 µmol of photons m^{-2} s^{-1} < 700 nm	(Onami et al., 2025)
Chromera velia	SAR	Marine, coastal waters, endozoic (inside corals)	Red shifted antenna complexes: Red-Chromera light harvesting (Red-CLH). Red-CLH complexes is assembled starting from a 17kDa polypeptide, with other LHC proteins. Red-CLH functionally connect with PSII.	Red monochromatic light at 635 nm	(Kotabová et al., 2014; Bína et al., 2014)
Phaeodactylum tricornutum	SAR	Marine, shallow coastal waters	Red shifted antenna complex constituted by an oligomer of Lhcf15, associated with PSII. Thylakoid membranes aggregate in superstacks.	Incandescent light with λ > 650 nm	(Fujita & Ohki, 2004; Herbstová et al., 2015; Bína et al., 2016; Herbstová et al., 2017)
Trachydiscus minutus	SAR	Freshwater, fishpond	Aggregation of >10 LHC monomers, forms rVCP complex associated with both PSs	Incandescent light with λ > 650 nm	(Bína et al., 2019; Litvín et al., 2019)
***Eustigmatophyceae* sp. FP5**	SAR	Freshwater, water sample from circulating water system partially shaded	VCP-like complex with shifted absorption. The antenna system is constituted by monomers where protein environment shift chlorophyll's absorption toward long wavelengths.	Monochromatic FR light at 740 nm	(Wolf et al., 2018; Niedzwiedzki et al., 2019)

Fig. S1 Light spectra used in this work. a) SOL, solar-like spectrum, b) FR, far-red light spectrum.

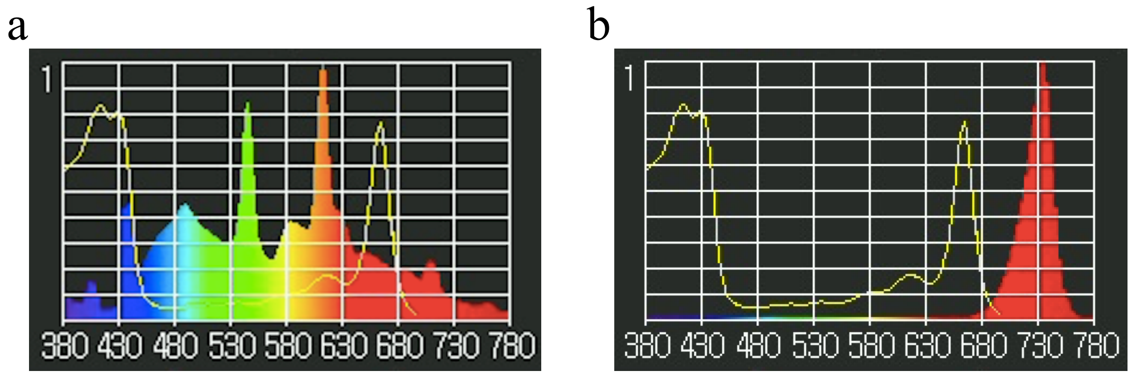

Table S2 Distribution of light in the two spectra used in this work: SOL (solar-like) and FR (far-red light). In the table is indicated the total amount of µmol of photons $m^{-2}s^{-1}$ provided, the amount this intensity provides in the wavebands far-red (700-780 nm), red (600-700 nm), green (500-600 nm), blue (400-500 nm), UV (380-400 nm), and the total amount of VIS (visible) light is reported.

	**SOL**	**FR**
**Total light (380-780 nm)** µmol of photons $m^{-2}s^{-1}$	25	25
**Far Red light (700 -780 nm)** µmol of photons $m^{-2}s^{-1}$	2.25	23.19
**Red light (600-700 nm)** µmol of photons $m^{-2}s^{-1}$	9.00	1.69
**Green light (500-600 nm)** µmol of photons $m^{-2}s^{-1}$	8.47	0.05
**Blue light (400-500 nm)** µmol of photons $m^{-2}s^{-1}$	5.02	0.05
**UV light (380-400 nm)** µmol of photons $m^{-2}s^{-1}$	0.26	0.02
**Total VIS light (380-700 nm)** µmol of photons $m^{-2}s^{-1}$	22.75	1.81

Fig. S2 Experimental setup for the reflectivity measurements. Yellow arrows highlight the light path. Created in BioRender. Liistro, E. (2025) https://BioRender.com/wpv1d4d

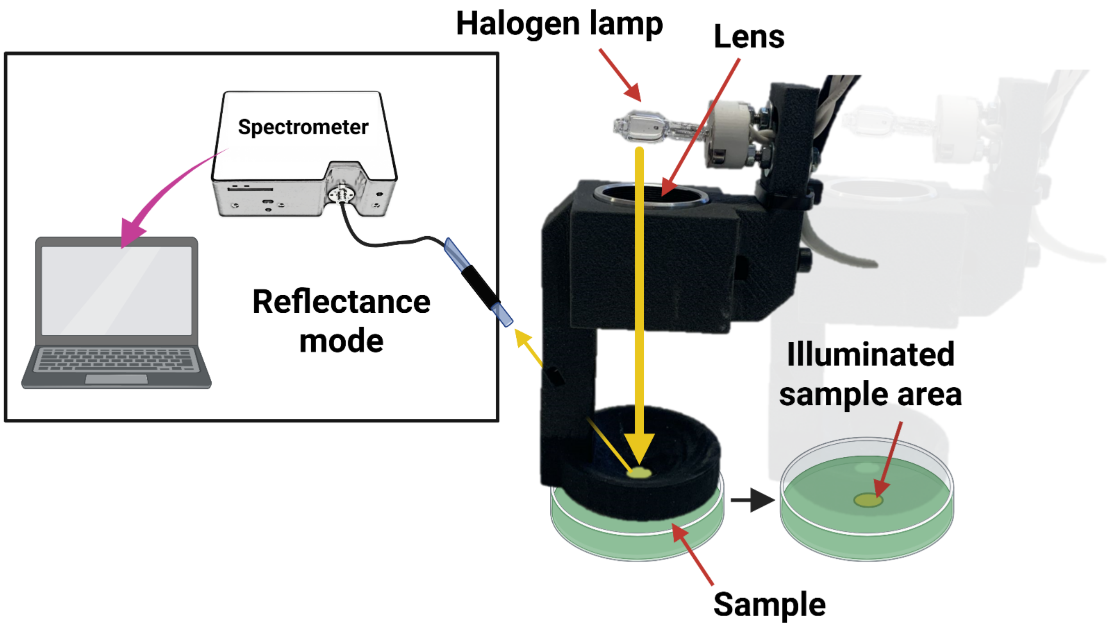

Fig. S3 Detection of *thylakoidal bodies* presence in SOL (a and d) and FR (b, c, e and f) acclimated cells. In micrographs presenting thin and distanced thylakoids stacks without consistent interconnections (a and d) *tb* were classified as “not detected”; in micrographs presenting thick and close thylakoids stacks with electron dense matter in between (★, b and e) *tb* were classified as “under construction”; in micrographs presenting big stacks interconnected with electrondense perpendicular bands (▲, c and f) *tb* were classified as “detected”. Scale bars correspond to 200 nm.

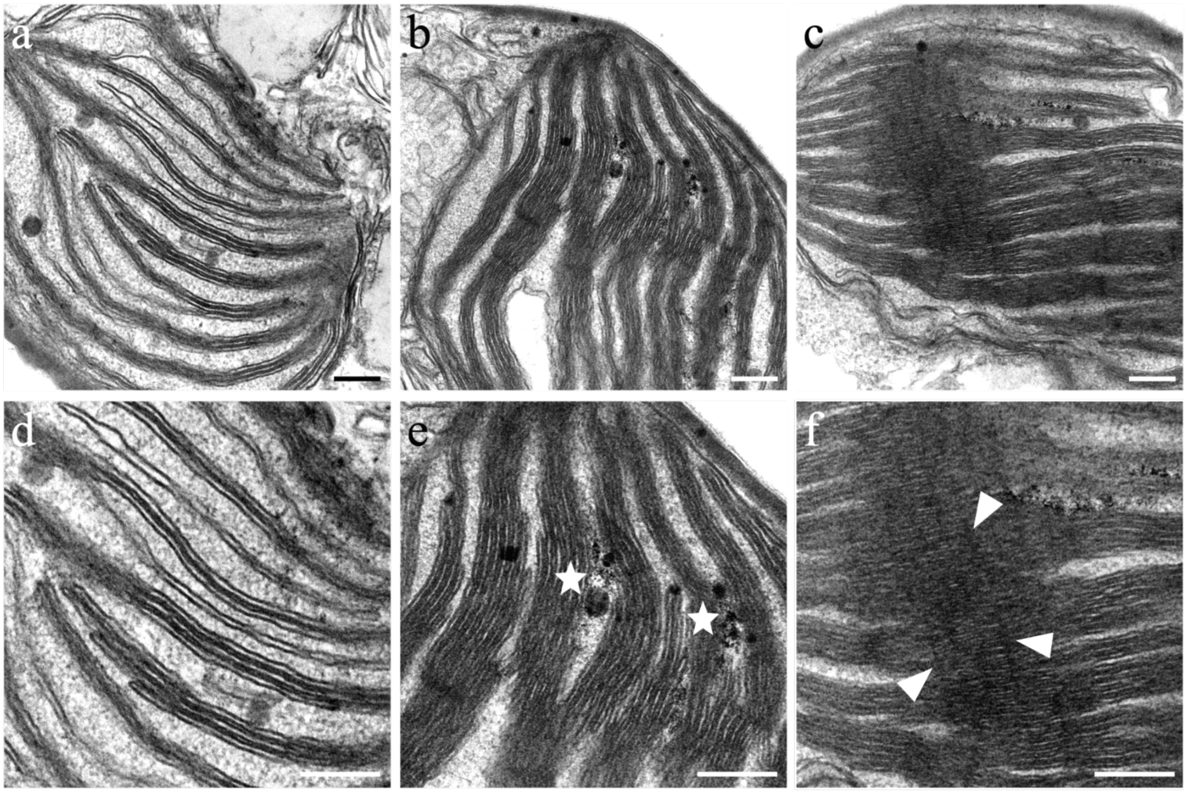

Fig. S4 . Pipeline of image processing. Original images (a) were binarized (b) then, with the "local thickness" tool a geometry-to-distance map (c) was obtained, and the local thickness was calculated (d). Scale bar corresponds to 500 nm.

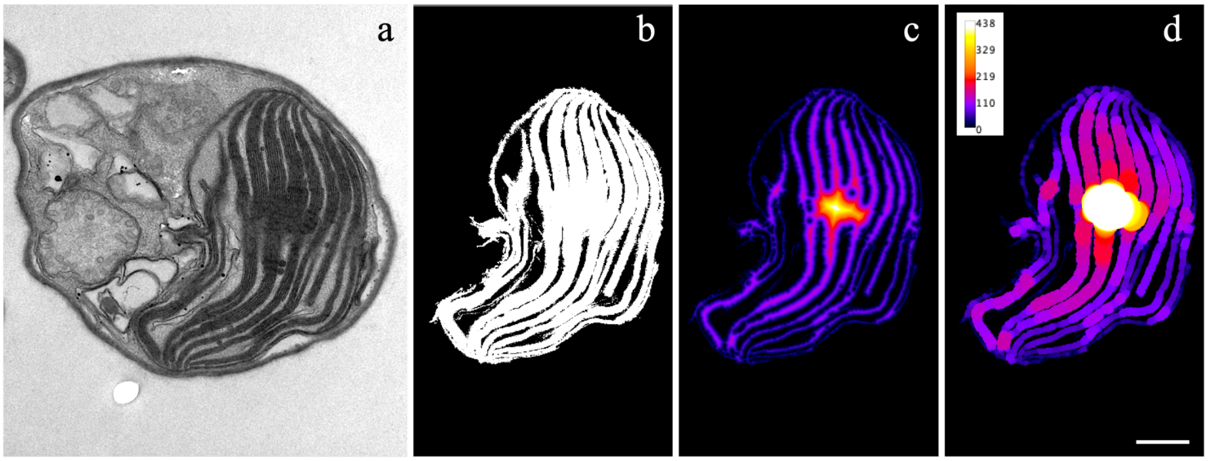

Fig. S5 Appearance of filtered cultures at equal cell concentration, acclimated to solar (a) and far-red (b) light.

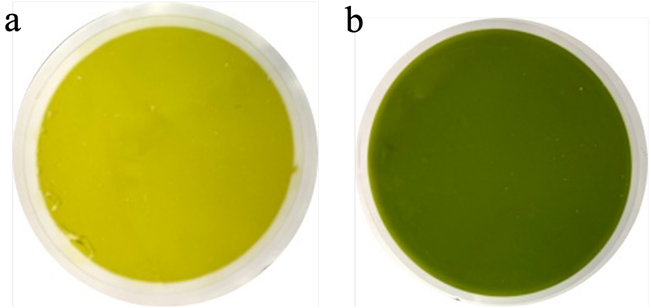

Fig. S6 Western blot analysis of FR-acclimated and SOL-acclimated *N. gaditana* cells. The numbers on top indicate µg of Chl *a* in the loaded sample. VCP = violaxanthin–chlorophyll-a-binding protein; D2 = photosystem II D2 protein; PsaA = photosystem I P700 chlorophyll *a* apoprotein A1; RbcL = Ribulose-1,5-bisphosphate carboxylase/oxygenase large subunit; LHCX1 = light-harvesting complex X1 protein. SOL: solar-like light; FR: far-red light.

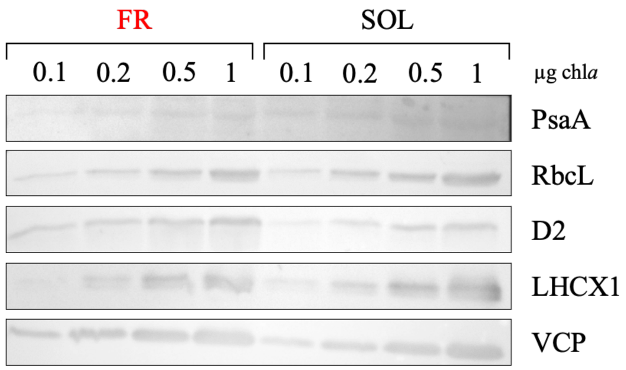

Fig. S7 P700 oxidation and reduction kinetics upon treatment with saturating light in absence (black line) and presence of DCMU (red line) or DCMU and DBMIB (blue line) in SOL-acclimated (left) and FR-acclimated (right) cells. DCMU: 3-(3,4- dichlorophenyl)-1,1-dimethylurea; DBMIB: dibromothymoquinone.

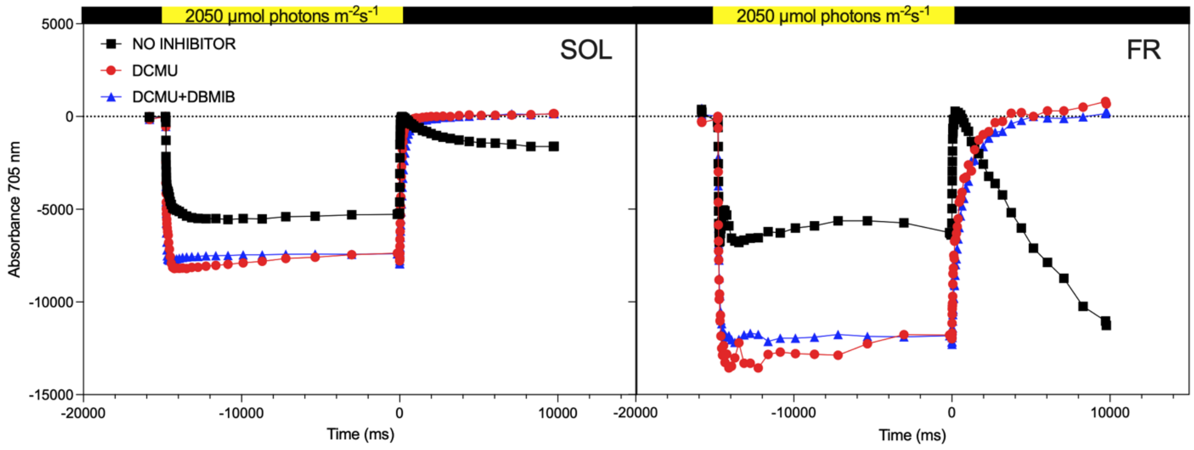

Fig. S8 Transmission electron micrographs of solar (SOL, left) and far-red (FR, right) light acclimated cells, with magnification on chloroplasts. Scale bars correspond to 500 nm.

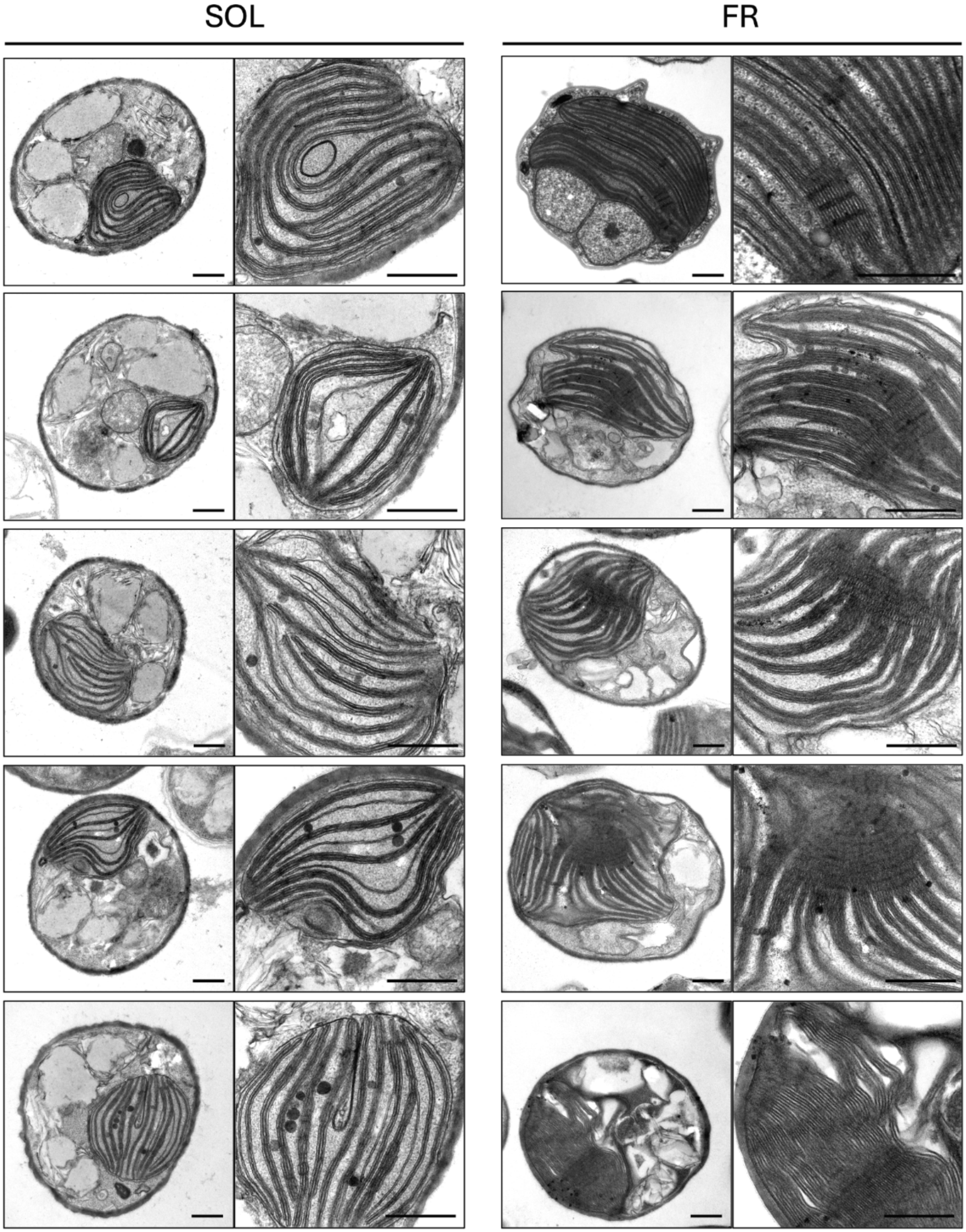

Supplement: Supplementary file 2 — Fig. S1 Light spectra employed in the work. Fig. S2 Experimental setup for the reflectivity measurements. Fig. S3 Representative images as references for the detection of thylakoidal bodies. Fig. S4 Pipeline of image processing for the local thickness analysis. Fig. S5 Appearance of acclimated cultures. Fig. S6 Western blot analysis. Fig. S7 P700 oxidation and reduction kinetics. Fig. S8 Representative micrographs of cells. Table S1 Summary of the far‐red light using algae and relative strategies. Table S2 Repartition of light in the spectra used depending on the waveband. Please note: Wiley is not responsible for the content or functionality of any Supporting Information supplied by the authors. Any queries (other than missing material) should be directed to the New Phytologist Central Office. [file NPH-249-1785-s001.pdf]
